# Integrated flavoromics and metabolomics characterize flavor and chemical composition differences in American ginseng–ripe Pu-erh tea blends

**DOI:** 10.3389/fnut.2026.1963132

**Published:** 2026-09-28

**Authors:** Man Zou, Ying Liu, Xiujuan Deng, Xin Fang, Yetao Xiao, Zuzhen Wang, Guangxing Li, Shuheng Zhang, Baijuan Wang, Xinghua Wang

**Affiliations:** 1 College of Tea Science, Yunnan Agricultural University, Kunming, Yunnan, China; 2 Yunnan Organic Tea Industry Intelligent Engineering Research Center, Kunming, China

**Keywords:** American ginseng, flavoromics, medicine and food homology, quality evaluation, ripe Pu-erh tea

## Abstract

Pu-erh tea occupies an important position in tea consumption because of its distinctive flavor and potential health benefits; however, its flavor acceptability still has room for improvement. To enhance sensory quality and diversify product forms, this study investigated a blending system of American ginseng powder and ripe Pu-erh tea powder and compared the quality and chemical composition of samples with different blending ratios by integrating sensory evaluation, physicochemical analysis, flavoromics, and untargeted metabolomics. The results showed that SP2, consisting of 75% ripe Pu-erh tea powder and 25% American ginseng powder, achieved the highest overall sensory score among the experimental blends (SP1–SP3) and was characterized by an aged aroma accompanied by a chrysanthemum-like note, a mellow and full-bodied taste, and a pronounced sweet aftertaste. As the proportion of American ginseng increased, the contents of caffeine and esterified catechins decreased, whereas the soluble sugar content increased. Tentatively identified 2-methylbutanal and (E)-2-octenal in SP2 were considered potential aroma-active candidates. A total of 1,171 tentatively annotated non-volatile metabolic features were obtained, with representative differential features involving flavonoids, phenylpropanoid-related compounds, and ginsenoside-related components. These compositional differences mainly reflected the respective contributions of the two raw materials and changes in their blending proportions, providing a reference for formulation optimization and quality evaluation of blended products.

## 1 Introduction

American ginseng is the dried root of *Panax quinquefolius* L., a plant belonging to the family Araliaceae. In traditional Chinese medicine, it is characterized as cool in nature, sweet and slightly bitter in taste, and non-toxic, and has traditionally been used to invigorate vital energy, nourish Yin, clear internal heat, and promote the secretion of saliva. It has a long history of use in China (1). Previous studies have shown that American ginseng is rich in diverse bioactive constituents, including ginsenosides, polysaccharides, flavonoids, and organic acids, and exhibits various pharmacological activities, such as blood glucose-lowering effect (2, 3), regulation of lipid metabolism (4), and antitumor activity (5). American ginseng has been widely incorporated into dietary therapies and functional food formulations. In 2023, American ginseng was officially approved in China for dual use as a food ingredient and a traditional Chinese medicinal material, further expanding its potential applications in food products (6).

Ripe Pu-erh tea is a distinctive post-fermented tea produced from sun-dried green tea made from the Yunnan large-leaf tea cultivar through a specific fermentation process. Its unique pile-fermentation process imparts a mellow and smooth taste as well as a characteristic aged aroma (7). During fermentation, complex chemical transformations and microbial activities markedly alter tea polyphenols, amino acids, and aroma-related compounds. Compared with other tea types, ripe Pu-erh tea is characterized by a mellow taste, relatively mild sensory properties, and a variety of potential health-promoting effects. Its major bioactive constituents include tea polyphenols, tea pigments, and alkaloids (8), which have been associated with multiple biological activities, including antioxidant and antitumor effects (9), blood glucose-lowering activity (10), as well as lipid-lowering and anti-obesity effects (11).

Against the backdrop of growing consumer demand for health-oriented products, increasing attention has been directed toward the potential roles of foods and beverages in disease prevention and daily health maintenance (12). Blending traditional tea with plants that are traditionally used as both food and medicine has therefore emerged as an important strategy for tea product innovation. American ginseng and ripe Pu-erh tea exhibit complementary characteristics in terms of both flavor and functional constituents. American ginseng is rich in ginsenosides and flavonoids, whereas ripe Pu-erh tea is characterized by abundant tea polyphenols, fermentation-derived products, and a distinctive aged aroma. However, current studies on American ginseng–ripe Pu-erh tea blends have mainly focused on sensory evaluation or the analysis of individual components, while systematic understanding of the compositional differences in volatile aroma compounds and non-volatile chemical constituents across different blending ratios and their associations with overall quality characteristics remains limited. Therefore, this study investigated American ginseng powder and ripe Pu-erh tea powder blended at different ratios. By integrating sensory evaluation, physicochemical analysis, volatile flavoromics, and untargeted metabolomics, we systematically characterized differences in sensory quality, volatile aroma compounds, and non-volatile chemical composition among blends with different ratios and further examined the relationships between these compositional characteristics and overall quality. This study aimed to provide a theoretical basis for optimizing the blending ratio and evaluating the quality of American ginseng–ripe Pu-erh tea blends.

## 2 Materials and methods

### 2.1 Materials and reagents

#### 2.1.1 Sample preparation and formulation screening

The experimental materials included ripe Pu-erh tea and American ginseng, both provided by Yunnan China Tea Co., Ltd., with each type of raw material originating from the same commercial batch. The samples were ground, sieved, sealed, and stored protected from light until further use. Detailed information on the raw materials, including harvest year, geographical origin, and production or fermentation conditions, is provided in Table 1. Prior to the integrated analyses of volatile flavoromics and untargeted metabolomics, preliminary screening experiments were conducted to determine the appropriate American ginseng material and range of blending ratios. A total of 11 samples were included in the preliminary screening (Table 2), comprising three American ginseng materials (Northeastern ginseng rootlets, Northeastern diced ginseng, and Wendeng diced ginseng), ripe Pu-erh tea, American Ginseng Pu-erh Xiaolicha (a commercial small-granule tea product), and blends prepared using different American ginseng materials and addition ratios.

**Table 1:** Basic information on the experimental raw materials.

Raw material	Harvest year	Geographical origin	Production/fermentation conditions	Batch number	Moisture content (%)	Milling method	Sieve mesh size (mesh)	Particle size (μm)	Storage temperature (°C)
Ripe Pu-erh tea	2025	Menghai County, Xishuangbanna Dai Autonomous Prefecture, Yunnan Province	Produced by conventional pile fermentation; detailed fermentation parameters were controlled by the manufacturer	Not available	8.8	High-speed mechanical grinding	60	<250	20–25
Wendeng American ginseng	2025	Menghai County, Xishuangbanna Dai Autonomous Prefecture, Yunnan Province	Wendeng American ginseng was dried and cut into pieces without fermentation	Not available	7.5	High-speed mechanical grinding	60	<250	20–25
Northeast American ginseng fibrous roots	2025	Menghai County, Xishuangbanna Dai Autonomous Prefecture, Yunnan Province	Northeast American ginseng fibrous roots were dried without fermentation	Not available	9.1	High-speed mechanical grinding	60	<250	20–25
Northeast American ginseng root pieces	2025	Menghai County, Xishuangbanna Dai Autonomous Prefecture, Yunnan Province	Northeast American ginseng root pieces were dried and cut into pieces without fermentation	Not available	6.9	High-speed mechanical grinding	60	<250	20–25

**Table 2:** American ginseng materials and ripe Pu-erh tea blend formulations.

No.	Blending formulation	Sample code	Experimental stage
1	Ripe Pu-erh tea powder	RP	Preliminary Screening and Comprehensive Analysis
2	Wendeng American ginseng powder	WS	Preliminary Screening and Comprehensive Analysis
3	Northeast American ginseng fibrous roots powder	DSX	Preliminary Screening
4	Northeast American ginseng root pieces powder	DSD	Preliminary Screening
5	65% ripe Pu-erh tea powder and 35% Wendeng American ginseng powder	SP1	Preliminary Screening and Comprehensive Analysis
6	75% ripe Pu-erh tea powder and 25% Wendeng American ginseng powder	SP2	Preliminary Screening and Comprehensive Analysis
7	85% ripe Pu-erh tea powder and 15% Wendeng American ginseng powder	SP3	Preliminary Screening and Comprehensive Analysis
8	American Ginseng Pu-erh Xiaolicha	SP4	Preliminary Screening and Comprehensive Analysis
9	95% ripe Pu-erh tea powder and 5% Wendeng American ginseng powder	SP5	Preliminary Screening
10	85% ripe Pu-erh tea powder and 15% Northeast American ginseng fibrous root powder	SP6	Preliminary Screening
11	85% ripe Pu-erh tea powder and 15% Northeast American ginseng root piece powder	SP7	Preliminary Screening

The preliminary screening was conducted in two stages: raw material screening and blending-ratio screening. First, Northeast ginseng fibrous roots, Northeast ginseng root pieces, and Wendeng ginseng root pieces were individually blended with ripe Pu-erh tea at an addition level of 15%. Physicochemical parameters and major non-volatile constituents determined by high-performance liquid chromatography (HPLC) were compared to evaluate compositional differences among the American ginseng materials and their corresponding blends. Based on the screening results, Wendeng ginseng root pieces were selected for subsequent formulation experiments. Subsequently, four additional levels of Wendeng ginseng root pieces (5, 15, 25, and 35%) were evaluated. The physicochemical parameters and major non-volatile constituents of these blends were further compared to select representative formulations for subsequent comprehensive analyses.

Based on the results of the preliminary screening, Wendeng diced American ginseng was selected as the American ginseng material, and three addition ratios (15, 25, and 35%) were chosen to prepare the blends for subsequent analyses. A total of six representative samples were included in the formal experiment: ripe Pu-erh tea powder (RP), Wendeng diced American ginseng powder (WS), three samples prepared by physically blending ripe Pu-erh tea powder and Wendeng diced American ginseng powder at different ratios (SP1–SP3), and a commercial American ginseng Pu-erh tea product, American Ginseng Pu-erh Xiaolicha (SP4). The ingredients of SP4 included American ginseng (≥15%), ripe Pu-erh tea, chicory root, and chrysanthemum. Because SP4 contained additional plant ingredients besides American ginseng and ripe Pu-erh tea, a binary blending system consisting exclusively of American ginseng and ripe Pu-erh tea was further established to minimize potential interference from other ingredients when comparing sample quality and chemical composition, and to investigate the quality and compositional characteristics of samples prepared at different blending ratios. RP and WS were used as reference samples for the two major raw materials, respectively; SP1–SP3 represented laboratory-prepared binary blends, whereas SP4 served as a commercial product reference. The detailed composition and blending ratios of all samples are provided in Table 2.

#### 2.1.2 Chemicals and reagents

The reagents used in this study included 95% ethanol, Folin–Ciocalteu reagent, methanol, ninhydrin, sodium carbonate, gallic acid, ethyl acetate, sodium bicarbonate, potassium dihydrogen phosphate, disodium hydrogen phosphate, glutamic acid, anthrone, glucose, tin(II) chloride, crystalline aluminum chloride, and sodium chloride. All reagents were of analytical grade and were purchased from Sinopharm Chemical Reagent Co., Ltd. (China).

Reference standards for catechins and flavonoids, including catechin (C), epicatechin (EC), epicatechin gallate (ECG), epigallocatechin (EGC), epigallocatechin gallate (EGCG), gallic acid (GA), gallocatechin (GC), catechin gallate (CG), gallocatechin gallate (GCG), theophylline, quercetin, taxifolin, caffeine, rutin, luteolin, kaempferol, and myricetin, were purchased from sigma-Aldrich (USA). HPLC-grade methanol and acetonitrile were also obtained from sigma-Aldrich.

### 2.2 Instruments and equipment

The principal instruments used in this study included a UV–visible spectrophotometer (Model 756CRT, Shanghai Jinghua Science and Technology Instrument Co., Ltd., Shanghai, China), an electronic analytical balance (Model CP214, OHAUS Instruments Co., Ltd., Shanghai, China), a high-speed centrifuge (Model CT15RE, Hitachi, Tokyo, Japan), a PEN3 electronic-nose system (Airsense Analytics GmbH, Schwerin, Germany), and an Agilent 1200 HPLC system equipped with an InfinityLab Poroshell 120 EC-C18 column (4.6 mm × 100 mm, 2.7 μm; Agilent Technologies, Santa Clara, CA, USA). Volatile compounds were analyzed using a Pegasus BT 4D GC × GC–TOFMS system (LECO Corporation, St. Joseph, MI, USA), and non-volatile metabolic features were analyzed using a Vanquish UHPLC–Q Exactive MS system equipped with an ACQUITY UPLC HSS T3 column (2.1 mm × 100 mm, 1.8 μm; Thermo Fisher Scientific and Waters, USA).

### 2.3 Measurement indices and methods

#### 2.3.1 Sensory evaluation

Sensory evaluation was conducted with reference to GB/T 23776-2018, Methodology of Sensory Evaluation of Tea (13). The appearance of each sample was evaluated first. Subsequently, 3.00 g of sample was weighed into a 150 mL standard evaluation cup and infused with purified water at 100 °C for 5 min. At the end of the prescribed infusion time, the tea infusion was completely drained from the evaluation cup into the evaluation bowl to ensure full separation of the infusion from the sample. The liquor’s color, aroma, and taste were then evaluated. Aroma was assessed at different temperature stages, while taste evaluation was performed when the infusion temperature had decreased to approximately 50 °C. According to the above standard, appearance (a), liquor color (b), aroma (c), and taste (d) were each scored on a 100-point scale, and the overall sensory score was calculated using Equation 1:

Overall sensory score=10%×a+20%×b+35%×c+35%×d (1)

The sensory evaluation panel consisted of five doctoral students majoring in tea science or food-related disciplines, including three females and two males, aged 35–40 years. All panelists had experience with at least 20 tea sensory evaluation sessions. Each panelist evaluated all samples under identical conditions in two rounds. The first round was conducted to assess the overall sensory quality of the samples and to complete the sensory descriptions and scoring, while the second round was used to review the results of the first evaluation in order to improve the accuracy and consistency of the sensory assessment. Samples were presented in the same fixed order as that used for physicochemical analyses, namely RP, WS, DSX, DSD, and SP1–SP7, without randomization. To minimize possible carryover effects between adjacent samples, the panelists thoroughly rinsed their mouths with purified water after evaluating each sample. All sensory evaluations were conducted in a clean, quiet, well-ventilated, odor-free environment with uniform and appropriate lighting. Informed consent was obtained from all panelists before the sensory evaluation. For each sensory attribute, the scores from the five panelists were averaged, and the mean attribute scores were then used to calculate the weighted overall sensory score.

#### 2.3.2 Determination of non-volatile components

The contents of water extract, total free amino acids, and tea polyphenols were determined according to the Chinese National Standards GB/T 8305-2013, GB/T 8314-2013, and GB/T 8313-2018, respectively. These methods have been widely applied to the analysis of tea quality-related components (14, 15). All samples were ground, sieved, and thoroughly homogenized prior to analysis. The results for all parameters were corrected according to the moisture content of the samples and expressed on a dry matter basis.

Water extract content was determined according to GB/T 8305-2013. Briefly, 2.00 g of each sample was accurately weighed, mixed with 300 mL of boiling distilled water, and extracted in a boiling water bath for 45 min. After extraction, the extract was immediately filtered under reduced pressure while hot, and the residue was thoroughly washed with boiling distilled water. The filter paper together with the insoluble residue was then dried in an oven at 120 ± 2 °C to constant weight. The water extract content was calculated based on the sample mass, dry matter content, and mass of the insoluble residue.

Total free amino acid content was determined according to GB/T 8314-2013 using the ninhydrin colorimetric method. Briefly, 3.00 g of each sample was accurately weighed, mixed with 450 mL of boiling distilled water, and extracted in a boiling water bath for 45 min. After extraction, the extract was immediately filtered under reduced pressure while hot, and the residue was washed two to three times with hot distilled water. The filtrate and washings were combined, cooled to room temperature, and diluted to 500 mL with distilled water. Subsequently, 1.0 mL of the sample solution was mixed sequentially with 0.5 mL of phosphate buffer (pH 8.0) and 0.5 mL of 2% ninhydrin solution, followed by color development in a boiling water bath for 15 min. After cooling, the solution was diluted to 25 mL with distilled water and allowed to stand for 10 min. The absorbance was then measured at 570 nm, and a standard curve was established using theanine as the reference standard.

Tea polyphenol content was determined according to GB/T 8313-2018 using the Folin–Ciocalteu colorimetric method. Briefly, 0.200 g of each sample was accurately weighed, mixed with 5.0 mL of 70% methanol preheated to 70 °C, and extracted in a water bath at 70 °C for 10 min. After cooling, the extract was centrifuged at 3,500 r/min for 10 min, and the supernatant was collected. The residue was extracted once more under the same conditions, and the two supernatants were combined and diluted to 10 mL with 70% methanol. An appropriate aliquot of the extract was mixed with Folin–Ciocalteu reagent and sodium carbonate solution. After thorough mixing, the reaction was allowed to proceed in the dark for 1 h. The absorbance was measured at 765 nm, and a standard curve was established using gallic acid as the reference standard.

Total soluble sugar content was determined using the anthrone–sulfuric acid method (16). Briefly, 1.00 g of each ground and sieved sample was accurately weighed, mixed with 80 mL of boiling distilled water, and extracted in a boiling water bath for 30 min. Immediately after extraction, the extract was filtered, and the residue was washed several times with small volumes of boiling distilled water. The filtrate and washings were combined and diluted to 500 mL with distilled water. A single extraction was performed without centrifugation. An appropriate aliquot of the extract was mixed with anthrone–sulfuric acid reagent and heated in a boiling water bath for 7 min for color development. The mixture was then rapidly cooled to room temperature in an ice-water bath, and the absorbance was measured at 620 nm. The soluble sugar content was calculated using a glucose standard curve. Each sample was analyzed in triplicate.

Catechins and flavonoid components were determined by high-performance liquid chromatography (HPLC) using a previously published method for the analysis of tea quality-related components with appropriate modifications (17). Briefly, 0.2 g of each sample was accurately weighed and mixed with 5.0 mL of 70% methanol preheated in a water bath at 70 °C. After thorough mixing, the sample was extracted in a water bath at 70 °C for 10 min. The extract was then cooled to room temperature and centrifuged at 3,500 r/min for 10 min, and the supernatant was collected. The residue was re-extracted once under the same conditions. The two supernatants were combined, diluted to 10 mL with 70% methanol, thoroughly mixed, and filtered through a 0.45 μm membrane filter. The resulting filtrate was used as the stock sample solution. Subsequently, 2.0 mL of the stock solution was accurately transferred into a 10 mL volumetric flask, diluted to volume, thoroughly mixed, and filtered again through a 0.45 μm membrane filter. The resulting filtrate was used for HPLC analysis. Sample extracts were stored at 4 °C and analyzed within 24 h. Three independent test solutions were prepared for each sample and analyzed separately. Chromatographic analysis was performed using an Agilent 1200 high-performance liquid chromatography system (Agilent Technologies, Santa Clara, CA, USA) equipped with a variable wavelength detector (VWD) and a C18 column (4.6 mm × 100 mm, 2.7 μm; Agilent, Santa Clara, CA, USA). Mobile phase A consisted of an aqueous solution containing 0.261% phosphoric acid and 5% acetonitrile, while mobile phase B consisted of 80% methanol. The gradient elution program was as follows: 0–16 min, B increased linearly from 1 to 45%; 16–22 min, B increased linearly from 45 to 65%; 22–25.9 min, B was maintained at 65%; 25.9–29 min, B increased linearly from 65 to 100%; and 29–30 min, B was maintained at 100%. The flow rate was 0.8 mL/min, the column temperature was maintained at 35 °C, and the injection volume was 2 μL. Catechins were detected at 280 nm from 0 to 20 min, whereas flavonoids were detected at 360 nm from 20 to 30 min.

Compounds were identified by comparing their retention times with those of authentic standards, all of which had purities of ≥98%. Mixed standard solutions and sample solutions were analyzed under identical chromatographic conditions and at the same detection wavelengths. A chromatographic peak in a sample was identified as the corresponding compound when its retention time matched that of the respective authentic standard, with a retention-time deviation of no more than ±0.1 min.

#### 2.3.3 Determination of volatile compounds

Electronic-nose analysis was performed with appropriate modifications based on the method described by Jin et al. (18). A PEN3 electronic nose (Airsense Analytics GmbH, Schwerin, Germany) equipped with 10 metal oxide semiconductor (MOS) sensors (W1C, W5S, W3C, W6S, W5C, W1S, W1W, W2S, W2W, and W3S) was used, and the main selectivities of the sensors are listed in Supplementary Table S1. Briefly, 1.00 g of each sample was placed in a 100 mL Erlenmeyer flask, sealed with two layers of sealing film, and allowed to equilibrate in the headspace at room temperature for 30 min. The measurement parameters were set as follows: cleaning time, 60 s; zeroing time, 10 s; pre-sampling time, 5 s; measurement time, 120 s; sampling interval, 1 s; and sampling flow rate, 300 mL/min. Each sample was measured in triplicate, and the mean sensor response values within the stable interval of 78–80 s were used for subsequent analysis. Sensor responses were expressed as *G*/*G*_0_, where *G* and *G*_0_ represent the sensor conductivity under sample headspace and clean-air conditions, respectively.

Volatile compounds were analyzed by headspace solid-phase microextraction coupled with comprehensive two-dimensional gas chromatography–time-of-flight mass spectrometry (HS-SPME-GC × GC-TOFMS). Briefly, 0.50 g of each sample was accurately weighed into a 20 mL headspace vial, followed by the addition of 4 mL of saturated NaCl solution and 10 μL of deuterated 1-hexanol-d13 internal standard solution (1 mg/L). After sealing, the vial was equilibrated at 80 °C for 10 min. Headspace extraction was performed using a DVB/CAR/PDMS fiber (50/30 μm × 1 cm; Supelco, Bellefonte, USA). Prior to use, the fiber was conditioned at 270 °C for 10 min and subsequently exposed to the sample headspace at 80 °C for 25 min. After extraction, the fiber was transferred to the GC injection port and desorbed at 250 °C for 5 min. Following each injection, the fiber was reconditioned at 270 °C for 10 min. In addition, 10 μL of an n-alkane standard solution was subjected to the same incubation, extraction, and injection procedures for retention index calculation and compound identification. For HS-SPME, each prepared sample was placed in a 20 mL headspace vial, followed by the addition of 4 mL of saturated NaCl solution and 10 μL of internal standard. After equilibration at 80 °C for 10 min, the headspace volatile compounds were extracted at 80 °C for 25 min using a DVB/CAR/PDMS-coated SPME fiber (50/30 μm × 1 cm). The fiber was subsequently inserted into the GC inlet and thermally desorbed at 250 °C for 5 min.

GC × GC-TOFMS analysis was performed using a LECO Pegasus BT 4D system (LECO, St. Joseph, MI, USA) equipped with an Agilent 8890A gas chromatograph, a dual-stage modulator, and a time-of-flight mass spectrometer. The first-dimensional column was a DB-Heavy Wax column (30 m × 0.25 mm × 0.50 μm), and the second-dimensional column was an Rxi-5Sil MS column (2 m × 0.15 mm × 0.15 μm). High-purity helium (>99.999%) was used as the carrier gas at a constant flow rate of 1.0 mL/min. The initial temperature of the first-dimensional column was 50 °C and was held for 2 min, then increased to 230 °C at 5 °C/min and held for 5 min. The second-dimensional column temperature was maintained 5 °C above that of the first-dimensional column throughout the analysis, while the modulator temperature was maintained 15 °C above that of the second-dimensional column. The modulation period was 6.0 s, and the injector temperature was set at 250 °C. The TOFMS was operated in electron ionization (EI) mode at 70 eV. Both the ion source and transfer line temperatures were maintained at 250 °C. Mass spectra were acquired over an m/z range of 35–550 at an acquisition rate of 200 spectra/s (19, 20).

Raw GC × GC-TOFMS data were processed using Chroma TOF software. Volatile compounds were annotated by combining mass spectral matching with retention index (RI) information. Mass spectra were matched against the NIST 2020 mass spectral library and an in-house database established by the analytical platform, with a minimum spectral similarity threshold of 700. Experimental RIs were calculated using C7–C30 n-alkanes and compared with reference RIs in the database (Lib_RI) as supplementary evidence to improve the confidence of compound annotation. For compounds lacking reference RI information or further confirmation with authentic standards, the identification was regarded as tentative. To evaluate the stability and repeatability of the HS-SPME-GC × GC-TOFMS analytical process, pooled quality control (QC) samples were included. The pooled QC sample was prepared by thoroughly mixing equal-mass portions of all study tea samples. For each QC analysis, 0.50 g of the pooled sample was weighed and subjected to the same sample preparation, HS-SPME extraction, and GC × GC-TOFMS conditions as the study samples. QC samples were periodically inserted throughout the analytical sequence to monitor the overall analytical stability, including headspace extraction, SPME fiber response, chromatographic separation, and mass spectrometric detection. Procedural blanks were also included. No tea sample was added to the blank, while all other sample preparation and analytical procedures were identical to those used for the study samples. These blanks were used to monitor background signals and potential contamination originating from reagents, the SPME fiber, headspace vials, and the analytical system. Three replicate samples (*n* = 3) were analyzed for each group. The within-group coefficient of variation (CV) for each volatile compound was calculated as the standard deviation divided by the mean and multiplied by 100%. Only compounds detected in all three replicates were included in the summary statistics for CV.

Relative odor activity values (ROAVs) were used to screen potentially aroma-active volatile compounds. Volatile compounds were semi-quantified using deuterated 1-hexanol-d13 as the internal standard, and their semi-quantitative contents were calculated based on the peak-area ratio of each analyte to the internal standard. The results for solid samples were expressed in μg/g and converted to mg/kg for ROAV calculation. The compound with the highest *C_i_*/*T_i_* ratio in each sample was selected as the reference compound and assigned an ROAV of 100, while the ROAVs of the remaining compounds were calculated according to Equation 2 (21).

$${\text{ROAV}}_{i}=\frac{C_{i}}{C_{\max}}\times \frac{T_{\max}}{T_{i}}\times 100$$ (2)

where *C_i_* and *T_i_* represent the semi-quantitative content and odor threshold of compound *i*, respectively, both expressed in mg/kg; *C*_max_ and *T*_max_ represent the semi-quantitative content and odor threshold, respectively, of the compound with the highest *C*/*T* ratio in the sample. The odor thresholds, threshold-determination matrices, and literature sources used for each compound are provided in Supplementary Table S4 (47–57). Given the matrix dependence of odor thresholds, ROAVs in this study were used only as a screening indicator for potentially aroma-active compounds.

#### 2.3.4 Non-volatile metabolomics analysis

Untargeted metabolomics analysis of the tea samples was performed with appropriate modifications based on the methods described by Zelena et al. and Want et al. (22, 23). Non-volatile metabolites were analyzed using ultra-high-performance liquid chromatography–tandem mass spectrometry (UHPLC–MS/MS). Briefly, 0.20 g of each sample was accurately weighed into a 2 mL centrifuge tube, followed by the addition of 600 μL of methanol extraction solution containing 2-chloro-L-phenylalanine as an internal standard at a final concentration of 4 mg/L. The mixture was vortexed for 30 s. Steel beads were then added, and the samples were homogenized using a tissue grinder at 55 Hz for 60 s, followed by ultrasonic extraction at room temperature for 15 min. The extracts were centrifuged at 12,000 r/min for 10 min at 4 °C, and the supernatants were filtered through a 0.22 μm PTFE membrane filter and transferred into LC–MS vials. The filtered extracts were stored at −20 °C until UHPLC–MS/MS analysis.

Chromatographic separation was performed using a Thermo Vanquish ultra-high-performance liquid chromatography system (Thermo Fisher Scientific, USA) equipped with an ACQUITY UPLC® HSS T3 column (2.1 × 100 mm, 1.8 μm; Waters, Milford, MA, USA). The flow rate was 0.3 mL/min, the column temperature was maintained at 40 °C, and the injection volume was 2 μL. For positive ionization mode, mobile phase A2 consisted of 0.1% formic acid in water, and mobile phase B2 consisted of 0.1% formic acid in acetonitrile. The gradient elution program was as follows: 0–1 min, 10% B2; 1–5 min, 10–98% B2; 5–6.5 min, 98% B2; 6.5–6.6 min, 98–10% B2; and 6.6–8 min, 10% B2. For negative ionization mode, mobile phase A3 consisted of 5 mM ammonium formate in water, and mobile phase B3 was acetonitrile. The gradient elution program was as follows: 0–1 min, 10% B3; 1–5 min, 10–98% B3; 5–6.5 min, 98% B3; 6.5–6.6 min, 98–10% B3; and 6.6–8 min, 10% B3.

Mass spectrometric detection was performed using a Thermo Q Exactive mass spectrometer (Thermo Fisher Scientific, USA) equipped with an electrospray ionization (ESI) source, with data acquired in both positive and negative ionization modes. The spray voltages were set to 3.50 kV in positive mode and −2.50 kV in negative mode. The sheath gas and auxiliary gas were set to 40 arb and 10 arb, respectively, and the capillary temperature was maintained at 325 °C. For full-scan MS analysis, the scan range was m/z 100–1,000 with a resolution of 70,000. Tandem MS analysis was performed using higher-energy collisional dissociation (HCD), with the normalized collision energy set to 30 eV and the MS/MS resolution set to 17,500. Data-dependent MS/MS acquisition (Full MS-ddMS2) was employed, in which the 10 most intense precursor ions in each scan cycle were selected for fragmentation. Dynamic exclusion was enabled to minimize repeated MS/MS acquisition of the same precursors.

To evaluate the stability of the UHPLC–MS/MS analytical process and overall data quality, pooled quality control (QC) samples were included. The QC sample was prepared by mixing equal volumes of the extracts from all samples and was analyzed under the same UHPLC–MS/MS conditions as the study samples. Before analysis of the study samples, the QC sample was injected three consecutive times to equilibrate and stabilize the analytical system. Thereafter, one QC injection was inserted after every six study samples throughout the analytical sequence to continuously monitor instrument response and signal stability. Blank samples were also included to monitor background signals and potential contamination. The study samples were analyzed in a fixed group order, and the injection sequence was not randomized. During LC–MS analysis, the autosampler temperature was maintained at 8 °C. Analytical stability was evaluated based on the clustering of QC samples in principal component analysis (PCA) and the relative standard deviation (RSD) of individual features. The QC samples showed good clustering in the PCA score plot, and 78.8% of the detected features exhibited RSD values below 30%, indicating good analytical stability. Only features with RSD ≤ 30% in the QC samples were retained for subsequent data analysis, whereas features with RSD > 30% were excluded.

### 2.4 Statistical analysis

Data processing was performed using Microsoft Excel 2021. Orthogonal partial least squares discriminant analysis (OPLS-DA) was conducted using SIMCA 14.1. Statistical significance was analyzed using SPSS 26.0, with *p* < 0.05 considered statistically significant. Bar charts were generated using GraphPad Prism 10.1, and heatmaps were created using TBtools-II (24). Additional data visualization was performed using Origin 2024, MetaboAnalyst 6.0, and Chiplot (www.chiplot.online).

Raw UHPLC–MS/MS data from the metabolomics analysis were converted to mzXML format using MSConvert (v3.0.8789) from the ProteoWizard software package. The converted data were then processed using the XCMS package (v3.12.0) in R for peak detection, peak grouping, retention-time correction, peak alignment, and missing-peak filling. Peak detection was performed using the centWave algorithm with the parameters ppm = 15, peakwidth = c (5, 30), and mzdiff = 0.01, while peak grouping was conducted with bw = 2 and mzwid = 0.015. A feature matrix containing mass-to-charge ratio (m/z), retention time, and relative peak intensity was subsequently generated.

Only features detected in more than 50% of the samples were retained, and missing values were imputed with one-half of the minimum non-zero value of the corresponding feature. Blank samples were used to screen background signals and potential contaminants, while pooled quality control (QC) samples were used to evaluate analytical stability. Support vector regression (SVR) was further applied to correct systematic drift during the analytical process. Features with a relative standard deviation (RSD) ≤ 30% in the QC samples were retained for subsequent analysis. After quality control, the data were mean-centered and unit-variance scaled (autoscaled) prior to multivariate statistical analysis.

Differential metabolic features were mapped to KEGG reference pathways based on their KEGG Compound IDs. Because the samples contained components derived from tea, American ginseng, and other plant materials, organism-nonspecific KEGG reference pathways were used rather than pathways restricted to a single species. The background set consisted of all tentatively annotated metabolites that passed quality control and could be assigned valid KEGG Compound IDs, whereas the input set comprised the differential metabolic features identified in each pairwise comparison. Duplicate annotations sharing the same KEGG identifier were merged prior to analysis.

Pathway enrichment was assessed using a one-sided hypergeometric test, with a nominal *p* value <0.05 without correction for multiple testing used as the screening criterion for potentially enriched pathways. Because no multiple-testing correction was applied, the enrichment results were interpreted only in an exploratory manner and were not considered confirmatory evidence of significant pathway enrichment. Pathway impact was calculated based on relative betweenness centrality, defined as the sum of the node centralities of the matched differential metabolites divided by the sum of the node centralities of all metabolite nodes in the corresponding pathway. The KEGG results were used only to characterize the composition of compounds sharing common biosynthetic origins among different samples and do not indicate that the corresponding pathways were biologically active in the dried products.

## 3 Results

### 3.1 Screening of American ginseng materials and blending ratios

A total of 11 samples were included in the preliminary screening (Table 2). A stepwise screening strategy was adopted to sequentially evaluate the American ginseng materials and addition ratios. The screening was primarily based on sensory evaluation results, physicochemical indices, HPLC results, and electronic-nose response characteristics. Sensory and physicochemical data obtained within the same screening stage were analyzed by one-way analysis of variance (ANOVA), followed by Tukey’s multiple-comparison test (*p* < 0.05). The sensory evaluation results of the 11 samples are presented in Supplementary Table S2.

First, Wendeng diced American ginseng (WS), Northeastern ginseng rootlets (DSX), and Northeastern diced ginseng (DSD) were compared. The overall sensory scores of WS, DSX, and DSD were 87.1 ± 0.13, 86.1 ± 0.13, and 83.9 ± 0.30, respectively, with significant differences among the three samples (*p* < 0.05); WS received the highest score. The total flavonoid content of WS was 19.79%, which was higher than those of DSX (1.48%) and DSD (1.22%; Supplementary Table S3). The contents of rutin, luteolin, quercetin, and kaempferol in WS were 35.20, 14.42, 6.14, and 2.56 mg/g, respectively, whereas these compounds were present at lower levels or were not detected in DSX and DSD. Considering both sensory quality and the contents of characteristic compounds, WS was selected as the raw material for subsequent blending experiments.

The blends SP3, SP6, and SP7, each containing 15% American ginseng, were further compared. Their overall sensory scores were 86.4 ± 0.37, 84.9 ± 0.23, and 83.4 ± 0.23, respectively, with SP3 scoring significantly higher than SP6 and SP7 (*p* < 0.05). These results indicated that, at the same addition ratio, the blend prepared with WS exhibited relatively better sensory quality, further supporting the selection of WS as the preferred American ginseng material.

Building on these results, SP5, SP3, SP2, and SP1 were prepared with WS addition ratios of 5, 15, 25, and 35%, respectively. The overall sensory scores of the four samples were 85.6 ± 0.19, 86.4 ± 0.37, 88.4 ± 0.23, and 85.5 ± 0.28, respectively, with significant differences among the groups (*p* < 0.05). Among them, SP2 containing 25% WS had a significantly higher overall sensory score than the other three experimental blends and was characterized by an aged aroma accompanied by a chrysanthemum-like note, a mellow and full-bodied taste, and a distinct sweet aftertaste. When the WS addition ratio increased to 35%, the characteristic American ginseng flavor of SP1 became more pronounced; however, its stronger particulate sensation and darker liquor color resulted in a lower overall sensory score. SP5 exhibited a relatively weak American ginseng flavor, with a taste score of only 83 points, and showed no obvious improvement in sensory quality.

The physicochemical and HPLC results further reflected compositional changes among samples with different WS addition ratios. The soluble sugar content of SP5 was 7.45%, which was close to that of RP (7.00%) and lower than those of SP3, SP2, and SP1 (9.12, 9.90, and 13.19%, respectively). The rutin and caffeine contents of SP5 were 2.00 and 34.10 mg/g, respectively, which were also close to those of RP (1.90 and 35.34 mg/g, respectively). As the WS addition ratio increased from 5 to 35%, the rutin content increased from 2.00 to 5.83 mg/g, whereas the caffeine content decreased from 34.10 to 29.67 mg/g. These results indicated that, within the 15–35% addition range, the contents of these non-volatile components in the blends changed in a regular manner with increasing WS proportion.

Considering the overall sensory performance, changes in non-volatile components, and the gradient of addition ratios, SP5 was relatively similar to RP in terms of soluble sugar, rutin, and caffeine contents, while its expression of American ginseng flavor and improvement in sensory quality were limited. Its additive contribution was therefore considered relatively small, and SP5 was not included in the subsequent integrated analysis. SP3, SP2, and SP1 were ultimately retained to represent low, medium, and high WS addition levels of 15, 25, and 35%, respectively. Although SP1 had a relatively lower sensory score, it was retained as the high-addition sample to investigate the effects of a higher American ginseng proportion on the quality characteristics of the blends.

A total of six samples, namely RP, WS, SP1, SP2, SP3, and SP4, were ultimately selected for subsequent analyses. RP and WS served as the ripe Pu-erh tea matrix control and the American ginseng raw-material control, respectively; SP1–SP3 were experimental blends representing different WS addition levels; and SP4 was included as a commercial reference sample containing additional plant ingredients. Although SP4 exhibited the highest overall sensory score (90.8 ± 0.23), its ingredient composition and processing conditions differed from those of the laboratory-prepared binary blends. Therefore, SP4 was not included in the direct comparison used for experimental formulation optimization.

Based on the above results, SP2 was selected as the preferred formulation among the experimentally prepared blends. This conclusion was restricted to the comparison among SP1–SP3 and does not indicate that SP2 had the highest sensory score among all samples.

### 3.2 Effects of different blending ratios on the quality characteristics of American ginseng–ripe Pu-erh tea blends

The taste of tea infusion is jointly determined by multiple water-soluble constituents. Among them, tea polyphenols, soluble sugars, and free amino acids shown in Figure 1A reflect the overall basis of taste-related substances in the samples, whereas the contents of caffeine and individual catechins were obtained from the HPLC results shown in Figure 1B. Previous studies have shown that caffeine is an important contributor to the bitterness of tea infusion, while esterified catechins such as EGCG and ECG generally exhibit relatively strong bitterness and astringency. Non-esterified catechins, including EC and EGC, can also contribute to bitter and astringent sensations, although their astringency is relatively weaker. Soluble sugars and free amino acids may contribute to sweetness and mellowness, freshness, and overall taste balance of the tea infusion (25–27).

**Figure 1:**
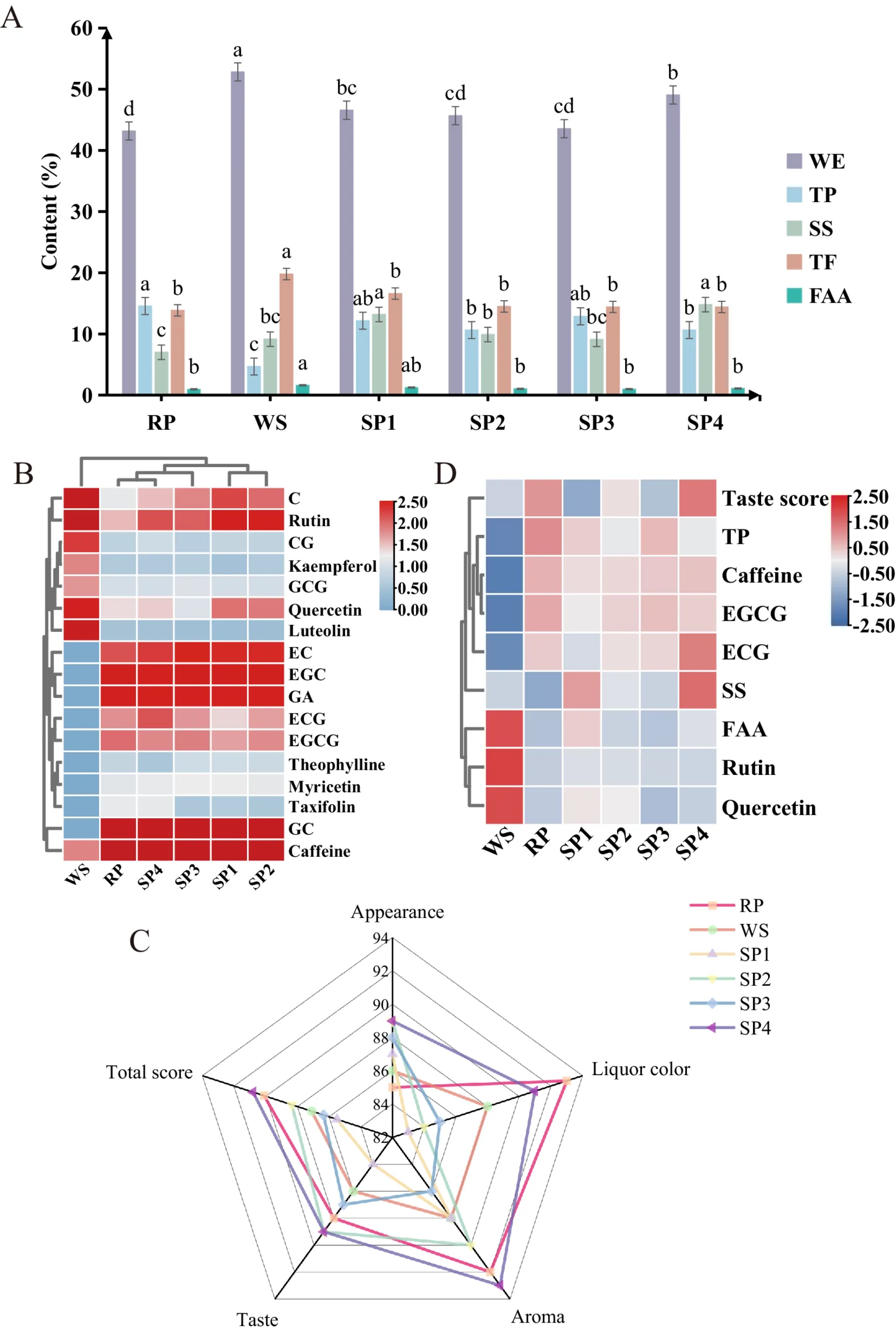
Sensory quality and non-volatile component characteristics of American ginseng–ripe Pu-erh tea samples with different blending ratios. **(A)** Contents of major physicochemical components; **(B)** standardized heatmap of components determined by HPLC; **(C)** radar plot of sensory attribute scores; **(D)** standardized heatmap of taste scores and major taste-related components. Data in panels B and D were standardized by individual variables, with colors ranging from blue to red indicating low to high relative levels of the same variable. Chemical indicators in panels A, B, and D were measured in triplicate (*n* = 3), whereas the sensory scores in panel C and the taste score in panel D were obtained from five panelists (*n* = 5). Data in panel A are presented as mean ± standard deviation, and different lowercase letters above the same indicator denote significant differences among samples (*p* < 0.05). WE, TP, SS, TF, and FAA represent water extract, total tea polyphenols, soluble sugars, total flavonoids, and free amino acids, respectively. For the HPLC components, C, EC, GC, and EGC represent catechin, epicatechin, gallocatechin, and epigallocatechin, respectively; CG, ECG, GCG, and EGCG represent catechin gallate, epicatechin gallate, gallocatechin gallate, and epigallocatechin gallate, respectively; GA represents gallic acid. RP and WS represent ripe Pu-erh tea powder and Wendeng American ginseng powder, respectively. SP1–SP3 are experimental blends of RP and WS at ratios of 65:35, 75:25, and 85:15 (w/w), respectively. SP4 is a commercial granulated tea reference sample containing American ginseng (≥15%), ripe Pu-erh tea, chicory root, and chrysanthemum.

The analysis of catechin and flavonoid components (Figure 1B) further showed that RP contained 35.34, 2.96, and 2.43 mg/g of caffeine, EGCG, and ECG, respectively, while its total tea polyphenol content reached 14.56%, collectively forming an important chemical basis for the bitterness and astringency of ripe Pu-erh tea. Compared with RP, the contents of caffeine, EGCG, and ECG in SP2 decreased to 30.47, 2.49, and 2.21 mg/g, respectively, whereas the soluble sugar content increased from 7.00 to 9.90%. These compositional changes may have partially reduced the bitterness and astringency associated with caffeine and esterified catechins while enhancing sweetness, mellowness, and overall taste balance, which was consistent with the sensory characteristics of SP2, including a relatively full-bodied taste and a certain degree of sweet aftertaste.

Among the experimental blends with different WS addition levels, as the proportion of WS increased from 15 to 35%, the caffeine content in SP3, SP2, and SP1 gradually decreased from 32.31 mg/g to 30.47 and 29.67 mg/g, respectively. The EGCG content decreased from 2.69 mg/g to 2.49 and 2.18 mg/g, while the ECG content decreased from 2.32 mg/g to 2.21 and 1.62 mg/g. Meanwhile, the soluble sugar content increased from 9.12 to 9.90 and 13.19%. Compared with SP3, SP2 contained lower levels of caffeine and esterified catechins and a higher level of soluble sugars. These changes may have been favorable for reducing bitterness and astringency and were consistent with its higher taste score (Figure 1C).

In contrast, although SP1 contained lower levels of caffeine and esterified catechins and a higher soluble sugar content, its total tea polyphenol content was 12.16%, which was higher than the 10.63% observed in SP2. In addition, the contents of rutin and quercetin in SP1 increased to 5.83 and 2.91 mg/g, respectively (Figure 1D). Some flavonoid components may contribute to the formation of bitterness and astringency, while the increased particulate and rough sensations associated with the higher WS addition level may also have contributed to the lower taste score of SP1 (Figure 1C).

The contents of EC and EGC changed relatively little among SP1–SP3, suggesting that the taste differences among samples with different WS addition ratios may be mainly related to the relative composition of caffeine, esterified catechins, total tea polyphenols, soluble sugars, and flavonoids derived from American ginseng, rather than being attributable to any single compound. Therefore, the relatively favorable taste profile of SP2 may result from a more appropriate compositional balance among tea-derived bitter and astringent compounds, soluble sugars, and American ginseng-derived flavonoids. These associations are inferred primarily from the known taste properties of these components and their changing trends among the samples, and do not establish a direct causal relationship between specific compounds and sensory attributes.

### 3.3 Differences in volatile compounds among American ginseng–Pu-erh tea blends with different blending ratios

Aroma quality is closely related to the composition, relative abundance, and odor thresholds of volatile organic compounds, and changes in the proportions of different classes of volatile components may collectively influence the overall odor characteristics of the samples (28, 29). In this study, untargeted volatile profiling of the six sample groups was performed using GC × GC–TOFMS, and a large number of volatile features were obtained after peak extraction, spectral library matching, and retention index comparison. Among the volatile features detected in all three replicates of the same sample, 59.30–71.16% exhibited coefficients of variation (CVs) below 30% (Figure 2A). Because authentic standards were not used in this study to confirm retention times and mass spectral matches, all compounds described below were regarded as tentatively identified volatile compounds.

**Figure 2:**
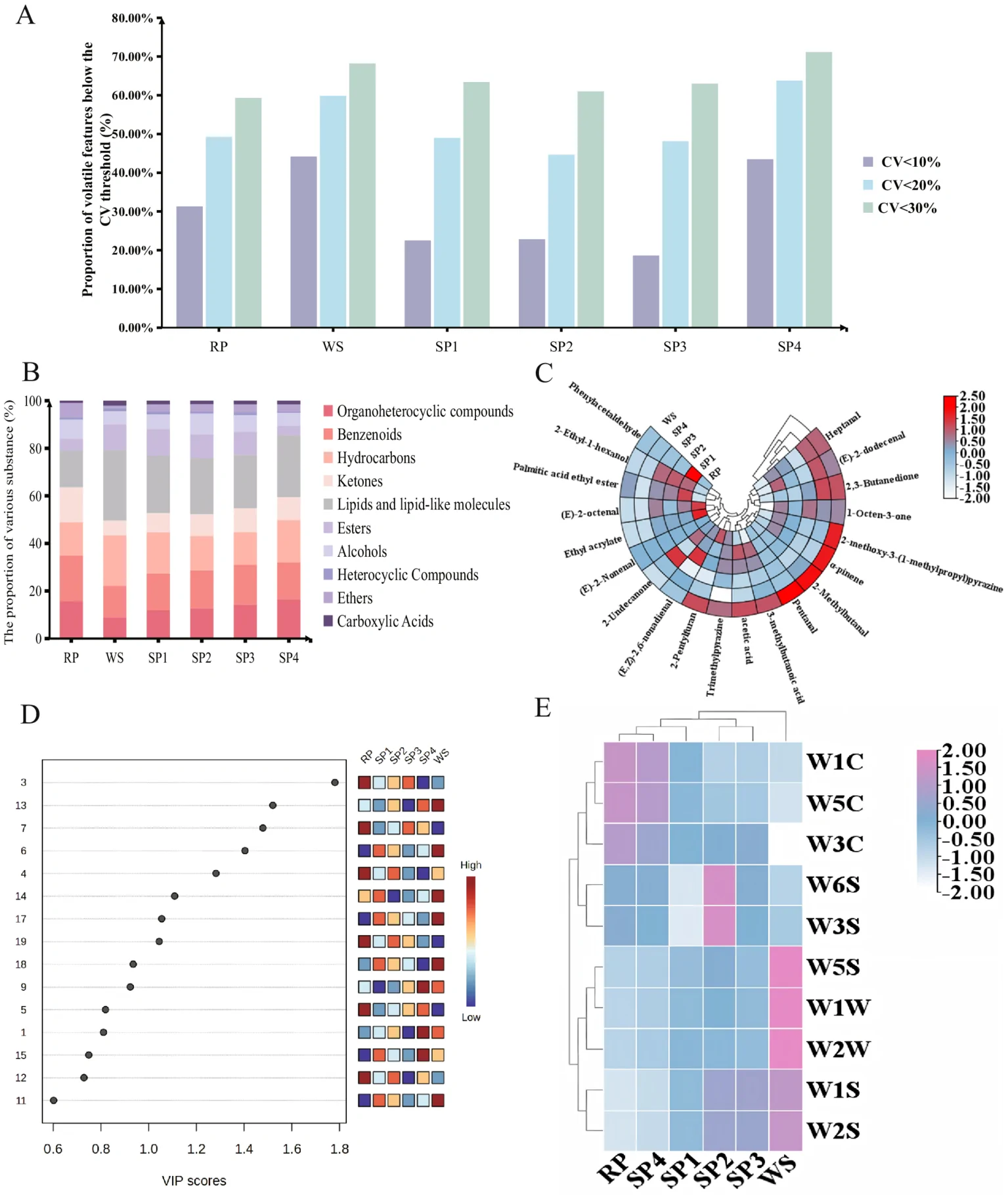
Volatile composition, aroma-active features, and electronic-nose responses of American ginseng–ripe Pu-erh tea samples with different blending ratios. **(A)** Cumulative proportions of volatile features commonly detected across three replicate measurements of each sample under different coefficient of variation thresholds (CV < 10%, CV < 20%, and CV < 30%); **(B)** relative abundance composition of different classes of volatile compounds; **(C)** circular heatmap showing the standardized relative abundances of 20 aroma-active compounds across different samples; **(D)** variable importance in projection (VIP) scores of the 20 aroma-active compounds and their relative abundance distributions across samples; **(E)** hierarchical clustering heatmap of the responses of 10 electronic-nose sensors. In panels C–E, colors from blue to red indicate low to high standardized relative levels. The definitions of RP, WS, and SP1–SP4 are the same as those in Figure 1. All measurements were performed in triplicate for each sample (*n* = 3). CV was calculated as the ratio of the standard deviation to the mean, i.e., CV (%) = SD/mean × 100%. In panel A, the denominator was the number of volatile features commonly detected in all three replicate measurements within each sample group.

The volatile compounds detected in the samples mainly included organic heterocyclic compounds, benzenoids, hydrocarbons, ketones, lipids and lipid-like molecules, esters, alcohols, ethers, and carboxylic acids (Figure 2B). In WS, lipids and lipid-like molecules and hydrocarbons accounted for relatively high proportions, at 29.61 and 21.35%, respectively. In RP, benzenoids and ketones were relatively abundant, accounting for 19.13 and 14.66%, respectively. As the WS addition ratio increased from 15 to 35%, the proportion of lipids and lipid-like molecules in SP3, SP2, and SP1 increased from 22.33 to 23.49 and 24.18%, respectively, while hydrocarbons increased from 13.74 to 14.45 and 17.36%. In contrast, organic heterocyclic compounds decreased from 14.22 to 12.73 and 11.93%, benzenoids decreased from 16.79 to 15.97 and 15.38%, and ketones decreased from 10.09 to 9.17 and 8.08%. These trends were generally consistent with the compositional differences between RP and WS, indicating that the volatile profiles of the blended samples mainly reflected the relative contributions of the two raw materials as their blending proportions changed.

For SP2, the proportions of the above compound classes were intermediate between those of SP1 and SP3, showing a relatively balanced compositional profile that was consistent with its sensory description of an aged aroma accompanied by a chrysanthemum-like note. However, the overall proportion of a given compound class does not directly represent its actual odor contribution and should therefore be evaluated together with odor thresholds and other relevant parameters. In addition, the mellow taste, sweet aftertaste, and persistent finish of SP2 were mainly associated with non-volatile taste-related components, indicating that volatile aroma compounds and non-volatile taste constituents jointly contributed to its overall flavor.

Given that only a limited number of volatile compounds can exert substantial effects on overall aroma, ROAV was further used to evaluate their potential odor contributions. Generally, compounds with ROAV ≥ 1 are considered key aroma-active compounds, those with 0.10 ≤ ROAV < 1 are regarded as important aroma modifiers, and those with 0.01 ≤ ROAV < 0.10 may exert potential effects (30, 31). In this study, among the 224 tentatively identified compounds with available odor-threshold data from the literature, 20 aroma-active volatile compounds were screened using ROAV > 0.10 as the criterion (Figure 2C) and were further compared in combination with OPLS-DA VIP values and one-way analysis of variance results (Supplementary Table S4). Among these, 10 compounds exhibited ROAV ≥ 1 in at least one sample.

In SP2, eight compounds exhibited ROAV ≥ 1, including (E)-2-nonenal, 2-methylbutanal, heptanal, (E)-2-octenal, 2,3-butanedione, 2-pentylfuran, 1-octen-3-one, and 2-undecanone. Their odor descriptions reported in the literature include fatty, green, malty, roasted, and mushroom-like notes. Among SP1–SP3, the ROAVs of 2-methylbutanal and 2-pentylfuran in SP2 were 11.03 and 3.86, respectively, both higher than those in SP1 (10.22 and 3.57) and SP3 (7.38 and 2.73). The ROAV of (E, Z)-2,6-nonadienal was also relatively high in SP2 (0.92). These results indicate that SP2 exhibited a distinct profile of potentially aroma-active compounds. However, the correspondence between these compounds and the sensory aroma characteristics of SP2 cannot be established from ROAV analysis alone. Detailed odor descriptions, threshold matrices, and literature sources are provided in Supplementary Table S4.

VIP (variable importance in projection) analysis further revealed the relative contributions of individual compounds to sample discrimination (Figure 2D). Ethyl acrylate exhibited the highest VIP value (1.78), followed by 2-methylbutanal (1.52), (E)-2-octenal (1.48), 2-methoxy-3-(1-methylpropyl) pyrazine (1.40), and trimethylpyrazine (1.28). Among these compounds, 2-methylbutanal and (E)-2-octenal simultaneously met the criteria of ROAV ≥ 1 and VIP > 1, indicating that they not only had relatively high potential odor contributions but also played important roles in differentiating the samples.

The electronic-nose results further confirmed differences in the overall odor profiles of the samples (Figure 2E). RP and SP4 showed relatively similar response patterns, while SP2 and SP3 clustered closely. WS exhibited higher responses in W5S, W1W, W2W, W1S, and W2S, whereas SP2 showed higher responses in W6S and W3S than SP1 and SP3. Taken together, the GC × GC–TOFMS, ROAV, VIP, sensory evaluation, and electronic-nose results indicated that SP2 exhibited a distinct volatile composition and relative odor-contribution pattern. Taken together, these results demonstrate differences in the volatile composition and relative odor-contribution profiles among the samples; however, they do not establish direct relationships between individual volatile compounds and the sensory aroma score of SP2.

### 3.4 Metabolite characterization of American ginseng–ripe Pu-erh tea blends with different formulations

#### 3.4.1 Integrated analysis of non-volatile metabolites in American ginseng–Pu-erh tea blends with different blending ratios

Metabolite annotation was performed by integrating the accurate mass-to-charge ratio of precursor ions, chromatographic retention time, and MS/MS fragmentation information. The mass tolerance for precursor-ion matching was set to 20 ppm. Molecular formulas were inferred based on accurate mass, mass error, and ion form, and were matched against HMDB, MassBank, KNApSAcK, ReSpect, LIPID MAPS, KEGG, and the PANOMIX in-house plant mass spectral library. The specific spectral-library sources recorded in the annotation results included BioDeepDB, mzVault, NaturalProducts, MoNA, METLIN, and mzkit_dia. The positive-ion forms involved in the annotation results included [M + H]^+^, [M + NH₄]^+^, [M + Na]^+^, [M + K]^+^, [M − H_2_O + H]^+^, [M − NH_3_ + H]^+^, [M − CO_2_ + H]^+^, [M − OH + H]^+^, and [2 M + H]^+^, whereas the negative-ion forms included [M − H]^−^, [M + HCOO]^−^, [M + HCO_3_]^−^, [M − H_2_O − H]^−^, [M − NH_3_ − H]^−^, and [2 M − H]^−^.

Metabolite annotation confidence was classified according to the Metabolomics Standards Initiative (MSI). Because authentic standards were not analyzed under identical analytical conditions to verify retention times and MS/MS spectra in this study, database-matched annotations were not regarded as definitive identifications. Entries with reliable MS/MS spectral library matches were assigned as putative annotations at MSI Level 2, whereas entries for which only the compound class or structural type could be inferred were classified as MSI Level 3 annotations (32). After quality control and annotation filtering, a total of 1,171 non-volatile metabolic features with tentative annotation information were obtained.

Six groups of samples were analyzed using UHPLC–Q Exactive MS-based untargeted metabolomics. After quality control, feature filtering, and database annotation, a total of 1,171 non-volatile metabolic features with tentative annotation information were obtained (Figure 3A). These features mainly included flavonoids, terpenoids and prenol lipids, phenols and phenylpropanoids, and alkaloids and N-containing compounds, comprising 233, 176, 156, and 142 features, respectively. These classification results reflect the overall composition of non-volatile constituents among the different samples.

**Figure 3:**
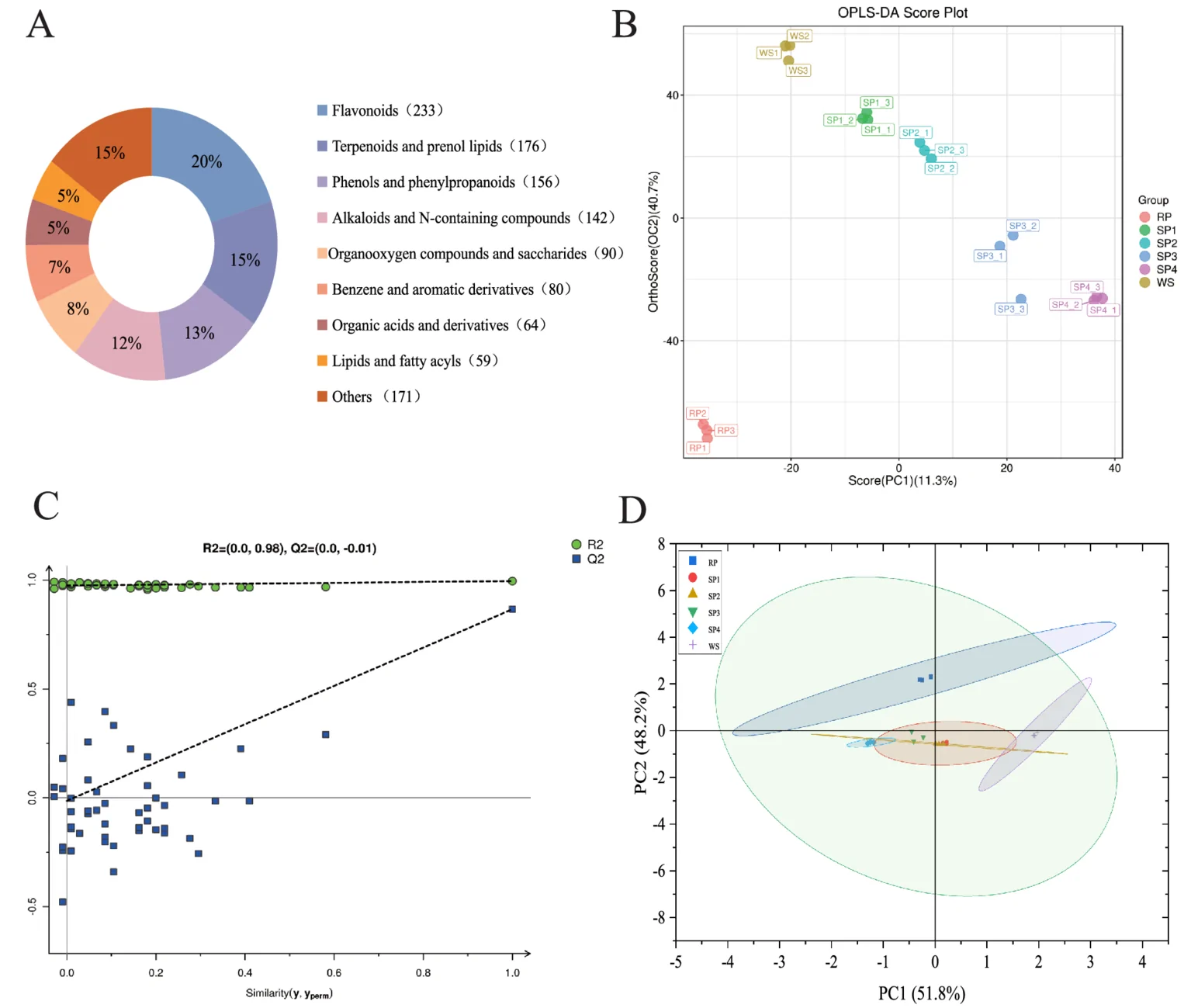
Composition and multivariate analysis of non-volatile metabolic features in American ginseng–ripe Pu-erh tea samples with different blending ratios. **(A)** Chemical class composition of non-volatile metabolic features; **(B)** orthogonal partial least squares-discriminant analysis (OPLS-DA) score plot; **(C)** permutation test results of the OPLS-DA model; **(D)** principal component analysis (PCA) score plot. Different colors in panels B and D represent different sample groups, with three replicate samples included in each group (n = 3). The definitions of RP, WS, and SP1–SP4 are the same as those in Figure 1.

To compare the non-volatile metabolic features among different samples, supervised analysis was further performed using OPLS-DA (Figures 3B,C). The model yielded *R*^2^*X*, *R*^2^*Y*, and *Q*^2^ values of 0.276, 0.988, and 0.735, respectively, indicating good model fit and a certain degree of cross-validated predictive ability. In the score plot, the different sample groups showed distinct distribution patterns, with replicates within each group generally clustering closely, although SP3 exhibited relatively greater within-group dispersion. Permutation testing showed that the *Q*^2^ values of all permuted models were lower than that of the original model, and the intercept of the *Q*^2^ regression line was −0.01, whereas the intercept of the *R*^2^ regression line was relatively high (0.98). Therefore, considering the small sample size and the potential risk of overfitting, OPLS-DA was used only as an exploratory discriminative analysis, and the results were interpreted in combination with PCA and univariate statistical analyses.

PCA showed that PC1 and PC2 explained 51.8 and 48.2% of the total variance, respectively (Figure 3D). Each group comprised three analytical replicates, and the samples from different groups exhibited distinct distribution trends in the score plot. RP and WS were located in separate regions, whereas SP1–SP3 were generally distributed between the two raw materials and showed a gradient pattern along PC1 that was associated with the blending ratio. SP1 and SP2 were positioned relatively close to each other, and their 95% confidence ellipses overlapped, indicating a certain degree of similarity in their overall non-volatile metabolite profiles; in contrast, SP1 remained clearly separated from WS. SP3 was located between SP2 and RP but showed relatively greater within-group dispersion. The commercial reference sample SP4 exhibited a distribution pattern distinct from those of the experimental blends. Hierarchical clustering analysis further reflected differences in non-volatile metabolic features among the different samples.

#### 3.4.2 Characterization of differential non-volatile metabolites in American ginseng–ripe Pu-erh tea blends with different formulations

Using VIP ≥ 1 (variable importance in projection), *p* < 0.05 (nominal unadjusted *p* value obtained from univariate statistical analysis), and |log_2_FC| ≥ 1 (FC, fold change) as the screening criteria, non-volatile metabolic features were compared between each blended sample and RP. The results showed that, compared with RP, 263, 242, 223, and 245 differential metabolic features were screened in SP1, SP2, SP3, and SP4, respectively. Overall, most differential features exhibited higher relative abundances in the blended samples than in RP. In SP2, 223 metabolic features showed higher relative abundances than in RP, whereas 19 showed lower relative abundances, with the former accounting for more than 90% of the total differential features in SP2. Because WS itself contains numerous compounds that are present at relatively low levels or are not detected in RP, these differences mainly reflect the contribution of WS incorporation and raw-material proportions to the chemical composition of the blended samples.

Hierarchical clustering analysis was further performed on the nine classes of differential non-volatile metabolite features to compare their relative abundance patterns among the samples (Supplementary Figure S1). The heatmap showed distinct relative-abundance patterns between RP and WS, whereas SP1–SP3 exhibited different combinations of features associated with the two raw materials. SP2 contained a relatively large proportion of high-abundance features, forming a corresponding high-abundance region in the heatmap. This distribution primarily reflected the contribution of compounds derived from WS after incorporation into the blending system.

Volcano plots of differential metabolic features, together with KEGG annotation and enrichment analyses, further revealed the compositional differences among samples (Figure 4). Differential features in SP1 and SP3 were mainly mapped to KEGG-associated compound sets, including flavonoid biosynthesis, phenylpropanoid biosynthesis, flavone and flavonol biosynthesis, and biosynthesis of various plant secondary metabolites. In addition to these categories, differential features in SP2 were also mapped to compound sets associated with linoleic acid metabolism and caffeine metabolism, indicating that the compositional differences between SP2 and RP involved multiple classes of compounds, including flavonoid- and phenylpropanoid-related compounds, lipid-related compounds, and alkaloids. As shown in Figure 4B, the pathway impact values for phenylpropanoid biosynthesis and flavone and flavonol biosynthesis in SP2 were 0.24 and 0.39, respectively. The latter showed a relatively higher topological impact value, indicating that the differential features mapped to this compound set occupied relatively more topologically influential positions within the KEGG reference network.

**Figure 4:**
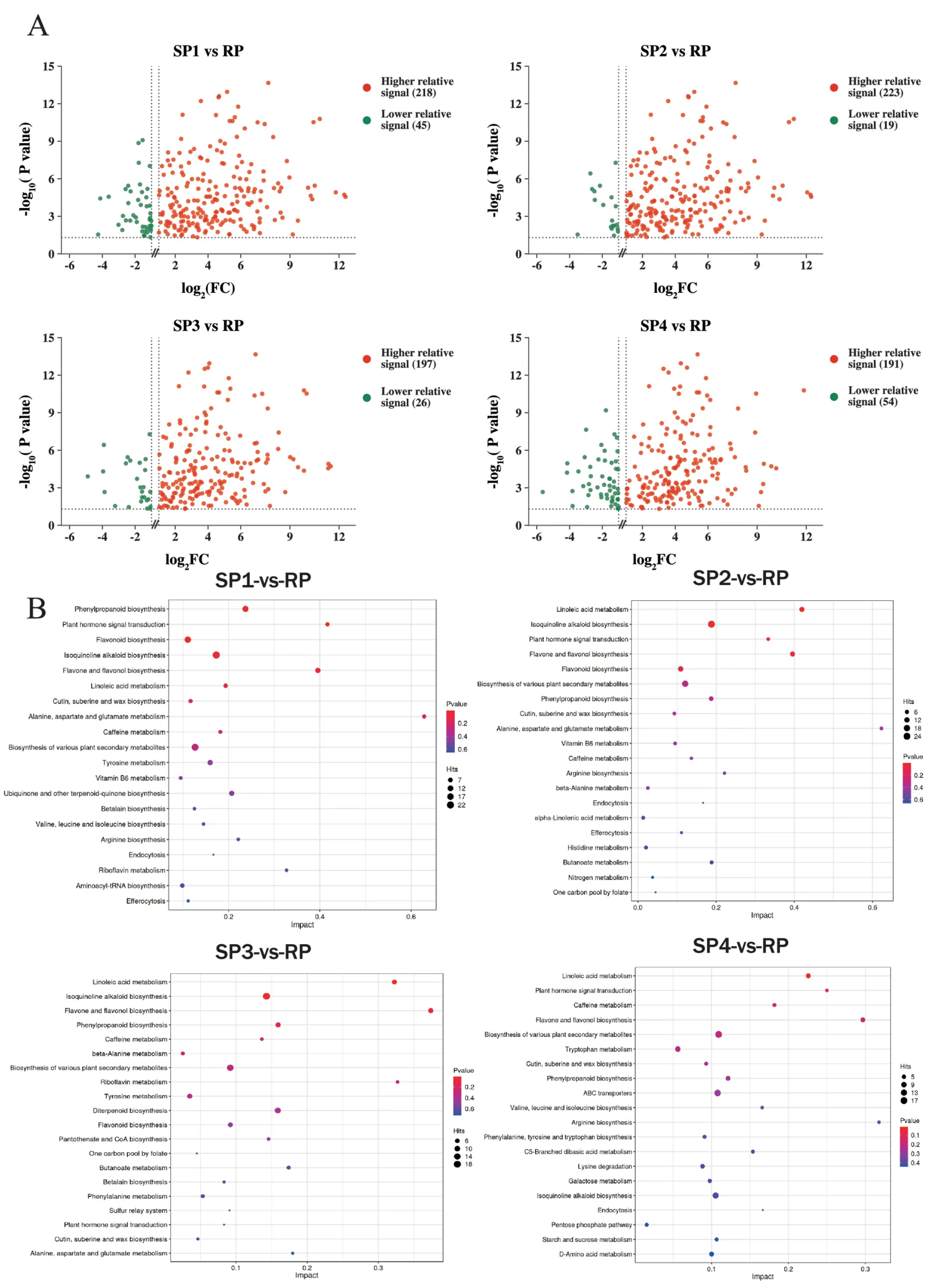
Differential non-volatile metabolic features and KEGG pathway analysis of American ginseng–ripe Pu-erh tea blends. **(A)** Volcano plots comparing SP1, SP2, SP3, and SP4 with RP, respectively. Red and green points indicate features with higher and lower relative abundances than RP, respectively. **(B)** KEGG pathway enrichment and topological analysis of differential features. Bubble size represents the number of hits, bubble color represents the *p* value, and the *x*-axis represents pathway impact. Differential features were screened using the criteria of VIP ≥ 1, *p* < 0.05, and |log_2_FC| ≥ 1. Each group contained three replicate samples (*n* = 3). Sample definitions are the same as those in Figure 1.

Taken together, the KEGG analysis revealed differences in the composition and relative abundance of compounds sharing common biosynthetic origins among the different samples. These differences in non-volatile constituents provide chemical information on the metabolic composition potentially associated with the mellow taste, sweet aftertaste, and prolonged finish of SP2. However, the causal relationships between these compositional features and specific sensory attributes remain to be further confirmed through targeted quantification and sensory validation.

#### 3.4.3 Compositional differences and KEGG mapping of key non-volatile metabolic features in American ginseng–ripe Pu-erh tea blends

Based on the screening of differential metabolic features and KEGG compositional mapping, representative features related to sample flavor and functional component composition were selected for comparison. These features were mainly mapped to KEGG compound sets associated with flavonoid biosynthesis, phenylpropanoid biosynthesis, flavone and flavonol biosynthesis, caffeine metabolism, and biosynthesis of various plant secondary metabolites. Because all samples were processed and dried products, these results only reflect compositional differences among compounds sharing common biosynthetic origins and do not indicate active biosynthetic or metabolic processes in the blending system. Compounds not verified using authentic standards and retention times were regarded as tentatively annotated, and their peak intensities were used only for relative comparisons among samples.

1. Flavonoid-Related Features

KEGG compositional mapping showed that 28 major differential features were mapped to compound sets associated with flavonoid biosynthesis and flavone and flavonol biosynthesis (Figure 5A). The latter had a pathway impact value of 0.3956, reflecting the topological positions of the matched features within the reference pathway network (Figure 4B). Features tentatively annotated as Kaempferol, Quercetin 3-(6′-malonyl-glucoside), and Apigenin 7,4′-dimethyl ether showed higher relative abundances in WS than in RP, while SP1–SP3 generally exhibited intermediate levels that varied with the proportion of WS added, indicating that these differences were mainly associated with raw-material contributions and blending ratios. The relatively low levels of some monomeric flavonoid-related features in RP may be associated with the pile fermentation undergone by ripe Pu-erh tea before blending, as well as with polyphenol oxidation and polymerization (8, 33).

**Figure 5:**
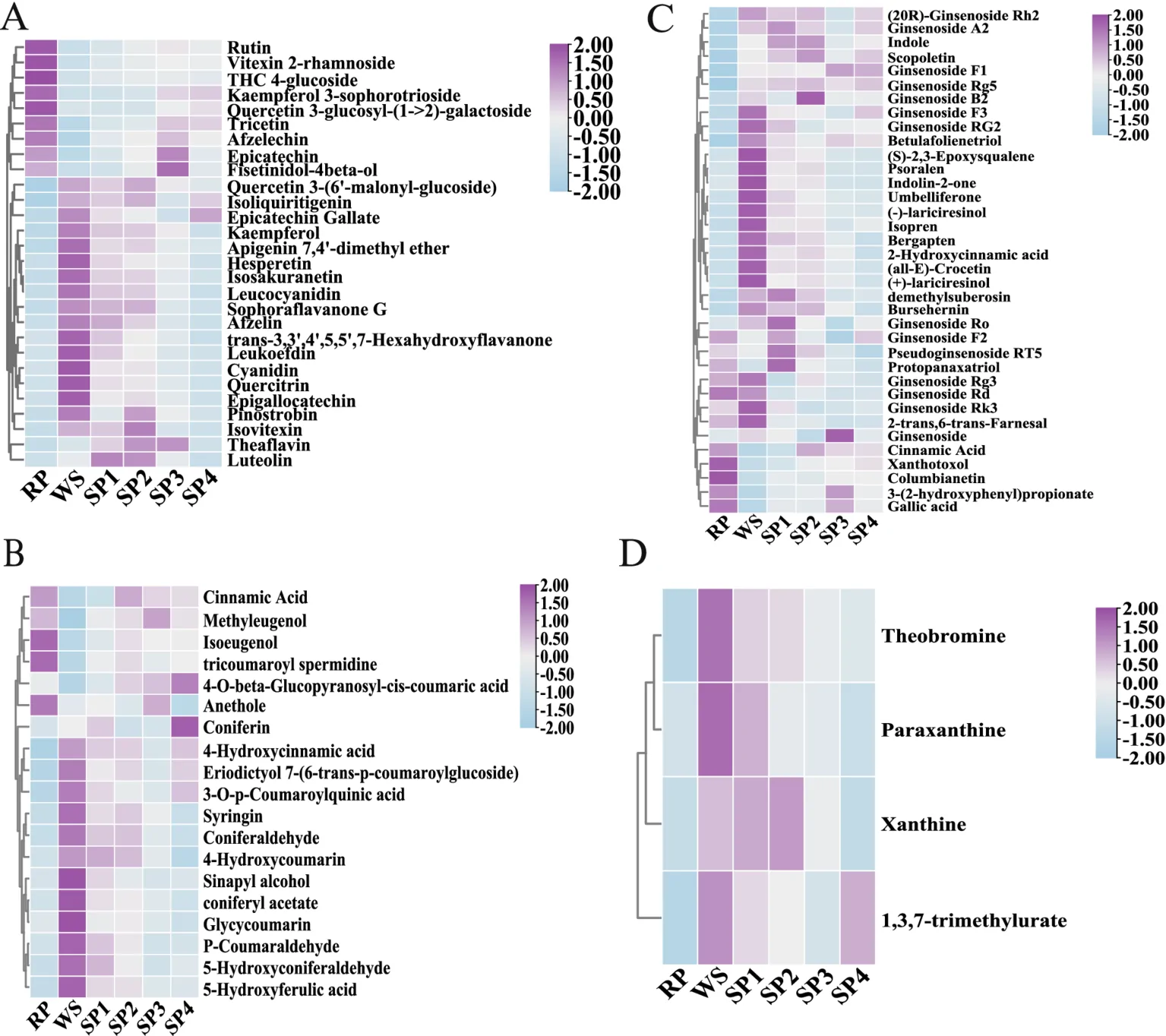
Relative abundance patterns of representative non-volatile metabolic features in American ginseng–ripe Pu-erh tea samples with different blending ratios. **(A)** Hierarchical clustering heatmap of tentatively annotated flavonoid- and flavonol-related features; **(B)** hierarchical clustering heatmap of tentatively annotated phenylpropanoid- and related phenolic features; **(C)** hierarchical clustering heatmap of tentatively annotated ginsenoside-related and associated features; **(D)** hierarchical clustering heatmap of tentatively annotated purine alkaloid features related to caffeine metabolism. Each group contained three replicate samples (*n* = 3). Heatmap data were *Z*-score standardized for each feature, with colors from blue to purple indicating low to high standardized relative abundance. The definitions of RP, WS, and SP1–SP4 are the same as those in Figure 1.

The feature tentatively annotated as theaflavin showed relatively high signal intensities in SP2 and SP3 but did not exhibit a clear gradient with the blending ratio. For compounds such as epicatechin gallate that had already been quantitatively determined by HPLC, their concentration changes were interpreted based on the HPLC results. Catechins and flavonol glycosides are associated with bitterness, astringency, and puckering sensations in tea infusions (34, 35). Therefore, the relatively higher signals of WS-related flavonoid features in SP1 may be associated with its more pronounced ginseng taste, particulate sensation, and rough mouthfeel, whereas the more moderate relative levels of these components in SP2 may help maintain taste body.

1. Phenylpropanoid-Related Phenolic Features

A total of 19 differential features were mapped to the compound set associated with phenylpropanoid biosynthesis, and the tentatively annotated phenylpropanoid- and related phenolic features showed distinct relative abundance patterns among the samples (Figure 5B). The pathway impact value was 0.2370 (Figure 4B). The feature tentatively annotated as 3-O-p-Coumaroylquinic acid showed an overall trend of WS > SP1 > SP2 > SP3 > RP, broadly consistent with the decreasing proportion of WS. The feature related to Eriodictyol 7-(6-trans-p-coumaroylglucoside) also exhibited relatively high abundance in WS and the blended samples, suggesting that it may have been mainly introduced by WS. In contrast, 4-O-β-Glucopyranosyl-cis-coumaric acid showed relatively low abundance in WS, while its levels in SP2 and SP3 were close to or slightly higher than those in RP, indicating that its distribution may have been influenced more strongly by tea-derived components and the blended matrix (Figure 5B).

Phenolic acids and flavonoid glycosides may contribute to sourness, bitterness, and astringency in tea infusions, although their actual sensory effects depend on their concentrations, sensory thresholds, and interactions with other components. The simultaneous presence of both WS-related and RP-related phenolic features in SP2 may provide a partial chemical explanation for its greater taste complexity and prolonged aftertaste.

1. Ginsenoside-Related Features

Figure 5C shows that multiple ginsenoside-related tentatively annotated features exhibited distinct relative abundance patterns across the six sample groups, including Ginsenoside F3, (20R)-Ginsenoside Rh2, Ginsenoside A2, Ginsenoside F1, Ginsenoside Rg5, Ginsenoside B2, Ginsenoside RG2, and Ginsenoside Ro. Among them, the features tentatively annotated as Ginsenoside F3 and (20R)-Ginsenoside Rh2 showed relatively low abundance in RP and higher levels in WS. Relatively high signals were also retained in SP1 and SP2, whereas SP3 showed generally lower levels, indicating a strong association of these features with WS-derived components.

American ginseng contains abundant and structurally diverse ginsenosides; therefore, these ginsenoside-related features may provide chemical information for evaluating the retention of American ginseng-derived components in the blended samples (1, 2, 4). However, authentic standards were not used in this study to verify retention times and MS/MS spectra. Accordingly, these compounds should be regarded only as tentatively annotated, and the specific isomers of Ginsenoside F3 and Rh2 cannot be confirmed, particularly the 20R configuration of Rh2. Ginseng and its ginsenoside constituents are generally associated with characteristic ginseng-like and bitter taste perceptions (36). The higher proportion of WS and stronger signals of some ginsenoside-related features in SP1 may be associated with its more pronounced ginseng-like taste. SP2, while retaining some ginsenoside-related components, exhibited a more balanced combination of ginseng-like aroma, aged aroma, and sweet aftertaste, suggesting that its overall sensory quality was more likely related to the relative composition of multiple tea-derived and ginseng-derived components rather than to any single ginsenoside.

1. Methylxanthine-Related Features

Features including xanthine, theobromine, paraxanthine, and 1,3,7-trimethylurate were mapped to the KEGG compound set annotated as caffeine metabolism (Figure 5D). Among them, theobromine, paraxanthine, and 1,3,7-trimethylurate showed relatively high signals in WS and some of the blended samples. Paraxanthine and 1,3,7-trimethylurate generally decreased across SP1–SP3 as the proportion of WS decreased.

The HPLC results showed that caffeine content decreased from 35.34 mg/g in RP to 32.31 mg/g in SP3 and 30.47 mg/g in SP2, more directly reflecting the proportional dilution of tea-derived caffeine following the addition of WS. The decrease in caffeine may have reduced tea-derived bitterness to some extent, whereas ginsenosides and phenolic components introduced by WS may have increased the complexity of ginseng-like taste and aftertaste. This was broadly consistent with the sensory characteristics of SP2, which was described as mellow, with a pronounced sweet aftertaste and a relatively long finish.

In summary, the differences in key non-volatile metabolic features were mainly determined by the intrinsic compositions of RP and WS, the blending ratios, and the prior pile-fermentation processing of ripe Pu-erh tea. Most WS-related features varied with the proportion of WS, reflecting the additive contribution of raw-material constituents. A small number of features that deviated from the blending-ratio gradient may also have been influenced by extraction efficiency, matrix-dependent ionization effects, and annotation uncertainty. SP2 retained both tea-derived caffeine and polyphenolic constituents as well as WS-derived ginsenoside-, flavonoid-, and phenolic-related features. The relative proportions of these components were consistent with the sensory evaluation results, showing a balanced combination of aged and ginseng aromas, a mellow and full-bodied taste, and a pronounced sweet aftertaste. These characteristics reflect a relative balance in the compositional contributions of the two raw materials rather than metabolic synergy or newly established metabolic regulation after blending. Because SP4 contained additional plant ingredients and was produced using a different processing method, it was included only as a commercial reference sample for separate comparison.

## 4 Discussion

### 4.1 Effects of blending ratios on sensory quality and the balance of taste-related components

The proportion of American ginseng added was not linearly related to the sensory quality of the blended samples. SP2 achieved an overall sensory score of 88.4 ± 0.23, the highest among the experimental blends SP1–SP3, and was characterized by a relatively balanced combination of aged and ginseng aromas, a mellow and full-bodied taste, and a pronounced sweet aftertaste. However, its score remained lower than those of RP (90.1 ± 0.27) and the commercial reference sample SP4 (90.8 ± 0.23). Therefore, SP2 should be regarded as the relatively preferred formulation within the experimental binary blending system rather than as having superior overall quality to RP or the commercial product.

As the proportion of WS increased, the contents of caffeine, EGCG, and ECG in the blended samples generally decreased, whereas the contents of soluble sugars, rutin, and several WS-related flavonoids increased. This trend was broadly consistent with the compositional differences between the two raw materials and mainly reflected the introduction of WS-derived constituents together with proportional changes in tea-derived components. Caffeine, esterified catechins, sugars, and flavonol glycosides can jointly influence bitterness, sweetness, mellowness, and astringency in tea infusions (25, 34, 35). However, SP1, which had the highest soluble sugar content, did not achieve the highest sensory score, indicating that an increase in soluble sugars alone was insufficient to improve overall taste quality. The relatively high levels of tea polyphenols, rutin, and quercetin in SP1 may be associated with its stronger astringency and rough mouthfeel, while suspended powder particles and differences in liquor color may also have influenced oral texture perception and sensory judgment (34, 35, 37). Therefore, the taste performance of SP2 was more likely related to the relative balance among multiple taste-related constituents and physical characteristics rather than to any single component.

Wu et al. (38) similarly observed an increase in soluble sugar content and an improvement in sweetness and mellowness after adding Polygonatum extract during black tea processing. However, in that study, the extract was incorporated during the rolling stage and subsequently underwent fermentation and drying, during which oxidation, degradation, and transformation may have occurred. In contrast, the present study involved the physical blending of already processed and dried RP and WS. Therefore, the compositional changes observed here are more appropriately interpreted as reflecting raw-material contributions and additive effects.

### 4.2 Relationship between volatile composition and overall aroma characteristics

As the proportion of WS increased, the relative proportions of lipids and lipid-like molecules and hydrocarbons increased, whereas those of benzenoids, ketones, and organic heterocyclic compounds decreased. These trends were broadly consistent with the compositional differences between RP and WS, indicating that the volatile composition of the blended samples was mainly influenced by the raw-material proportions. In SP2, the relative proportions of the major volatile classes were intermediate between those of SP1 and SP3, which was consistent with its relatively well-balanced aged and ginseng aroma characteristics. However, the relative abundance of a compound class does not directly reflect its actual odor contribution.

Eight tentatively identified compounds in SP2 exhibited ROAV values ≥ 1, among which 2-methylbutanal, 2-pentylfuran, and (E)-2-octenal showed relatively high potential odor contributions. These aldehydes, ketones, and furans have also attracted attention in previous studies on the aroma of tea and Pu-erh tea (28–30, 39, 40). Wu et al. (38) likewise identified some of these compounds in a black tea system supplemented with Polygonatum extract. This consistency suggests that these compounds may contribute to the overall odor profile of plant material–tea composite systems, although the overlap may also partly reflect the use of similar analytical platforms and ROAV-based screening approaches.

Ma et al. (40), using GC–O and related approaches, identified the key aroma-active compounds in Pu-erh tea as mainly including linalool, 1,2,3-trimethoxybenzene, 1,2,4-trimethoxybenzene, and α-ionone, which were not entirely consistent with the candidate compounds identified in the present study. These differences may arise from variations in raw-material composition, processing history, extraction methods, and the sources of odor-threshold data, and further indicate that instrumental responses and ROAV values cannot substitute for direct olfactory validation. The odor thresholds used in this study were derived mainly from aqueous matrices, whereas actual tea infusions represent a much more complex matrix. In addition, ROAV is a relative metric and may be influenced by the choice of reference compound and changes in the overall volatile composition. Therefore, compounds with ROAV ≥ 1 should be regarded only as potentially aroma-active compounds and cannot be considered as direct contributors to specific sensory attributes such as aged aroma, ginseng aroma, or “chrysanthemum-like” aroma. The electronic-nose results only support the presence of differences in the overall odor profiles among samples and cannot be used to confirm the sensory roles of specific compounds.

### 4.3 Differences in non-volatile metabolite composition and their raw-material origins

PCA showed that SP1–SP3 were generally distributed between RP and WS and exhibited a trend associated with the blending ratio, supporting the view that the non-volatile composition of the blended samples was jointly determined by the two raw materials. However, the confidence ellipses of SP1 and SP2 overlapped, and SP3 showed relatively greater within-group dispersion; therefore, SP2 should not be interpreted as representing a completely distinct metabolic type. The OPLS-DA model showed relatively high *R*^2^*Y* and *Q*^2^ values, and the *Q*^2^ values of the permuted models were lower than that of the original model. Nevertheless, the relatively high *R*^2^ intercept in the permutation test and the small sample size indicate that the risk of overfitting cannot be completely excluded. Therefore, the OPLS-DA model was used only as an exploratory discriminative tool, and its results were interpreted together with PCA and univariate statistical analyses based on nominal *p* values without multiple-testing correction (41).

Most differential metabolic features in the blended samples exhibited higher relative abundances than those in RP, mainly because WS contained numerous compounds that were present at relatively low levels or were not detected in RP. On the other hand, RP had already undergone pile fermentation prior to blending, during which catechins, phenolic acids, and other polyphenolic constituents may have experienced oxidation, polymerization, and degradation (8, 33, 42, 43). Changes in phenolic and flavonoid composition have also been reported when Polygonatum extract was added during tea processing (38) or when citrus and Pu-erh tea were subjected to co-fermentation (44). However, these systems involved subsequent fermentation or drying, and their transformation mechanisms cannot be directly applied to explain the physical blending system of finished products used in the present study.

Some metabolic features tentatively annotated as flavonoids, phenylpropanoid derivatives, and ginsenosides exhibited regular changes with increasing WS proportion, suggesting that they were likely derived primarily from the American ginseng material. Signals tentatively annotated as Ginsenoside F3 and (20R)-Ginsenoside Rh2 may serve as chemical indicators of the incorporation of WS-derived constituents into the blending system (1, 2, 4, 45, 46). For features such as tentatively annotated theaflavins that did not show a clear blending-ratio gradient, differences in matrix extraction, ionization effects, or annotation uncertainty represent plausible alternative explanations. For compounds such as caffeine, EGCG, and ECG, for which HPLC quantitative data were available, interpretation should primarily rely on the targeted measurements.

Accordingly, the KEGG results only reflect the compositional mapping of compounds sharing common biosynthetic origins among different samples, while pathway impact values represent the topological positions of the matched features within the reference network. Overall, the sensory performance of SP2 was consistent with its relative composition, in which both tea-derived constituents and WS-derived constituents were retained. However, because the measured values were not compared with theoretical mixture values, these observations can currently be interpreted only as blending-ratio-driven contributions of the two raw materials and do not provide evidence for non-additive effects or synergistic interactions.

### 4.4 Study limitations

The raw materials used in this study were derived from a limited number of production batches, and the three measurements conducted for each group mainly represented analytical replicates of samples from the same batch. Therefore, the present results may not fully capture the natural variability associated with different geographical origins, harvest years, and production batches. Sensory evaluation was conducted by only five panelists with relevant experience, and the samples were presented in a fixed order, which may have introduced order and carryover effects.

The GC × GC–TOFMS and UHPLC–MS/MS results were mainly based on untargeted tentative annotations and lacked confirmation using authentic standards and retention times. In addition, the ROAV values calculated using odor thresholds determined in water were not validated by GC–O, aroma recombination, or omission experiments. Meanwhile, the high-dimensional, small-sample design, together with the absence of CV-ANOVA and an external validation set, limited the generalizability of the OPLS-DA model (41). In addition, the feature-level univariate comparisons and KEGG pathway-enrichment analyses were based on nominal *p* values without correction for multiple testing. The candidate differential metabolic features and pathway associations identified in this study may include false-positive findings and should be interpreted only as exploratory. Furthermore, theoretical predicted values were not calculated based on the measured compositions of RP and WS and their blending ratios; therefore, it was not possible to determine whether the observed results deviated from simple additivity. Future studies should include independent raw-material and production batches and incorporate randomized sensory evaluation, theoretical mixture modeling, targeted quantification using authentic standards, statistical analyses with appropriate multiple-testing correction, as well as GC–O and aroma recombination experiments to validate potential differential metabolic features, candidate flavor compounds, and their sensory contributions.

## 5 Conclusion

This study demonstrated that the chemical composition of American ginseng–ripe Pu-erh tea blends varied with the blending ratio, whereas sensory quality did not improve continuously with increasing American ginseng addition. Under the raw-material and sensory evaluation conditions used in this study, SP2, consisting of 75% ripe Pu-erh tea powder and 25% American ginseng powder, was the relatively preferred formulation among SP1–SP3. It was characterized by an aged aroma accompanied by a chrysanthemum-like note, a mellow and full-bodied taste, and a pronounced sweet aftertaste; however, its overall sensory score remained lower than those of the ripe Pu-erh tea raw material and the commercial reference sample. As the proportion of American ginseng increased, the contents of caffeine and esterified catechins decreased, whereas the soluble sugar content increased. Volatile composition and flavonoid-, phenolic-, and ginsenoside-related features also showed blending-ratio-associated differences, mainly reflecting the compositional contributions of the two raw materials.

In SP2, tentatively identified 2-methylbutanal and (E)-2-octenal may be regarded as potential aroma-active compounds, while differences in non-volatile composition provide chemical compositional information that may help explain its taste characteristics. The KEGG results only reflect the mapping relationships between compound composition and reference pathways and do not indicate that metabolic activity was activated after blending. This study provides a reference for formulation selection and screening of candidate flavor compounds in binary blended teas; however, it does not demonstrate synergistic effects or enhanced biological activity. These conclusions should be further validated using additional independent batches, targeted quantification with authentic standards, and sensory verification.

## Data Availability

The raw data supporting the conclusions of this article will be made available by the authors, without undue reservation.

## References

[1] Yang L Hou A Zhang J Wang S Man W Yu H . Panacis Quinquefolii Radix: a review of the botany, phytochemistry, quality control, pharmacology, toxicology and industrial applications research progress. Front Pharmacol. (2020) 11:602092. doi: 10.3389/fphar.2020.602092, 33381041 PMC7768635

[2] Qi LW Wang CZ Yuan CS. Ginsenosides from American ginseng: chemical and pharmacological diversity. Phytochemistry. (2011) 72:689–99. doi: 10.1016/j.phytochem.2011.02.012, 21396670 PMC3103855

[3] Vuksan V Sievenpiper JL Koo VYY Francis T Beljan-Zdravkovic U Xu Z . American ginseng (*Panax quinquefolius* L.) reduces postprandial glycemia in nondiabetic subjects and subjects with type 2 diabetes mellitus. Arch Intern Med. (2000) 160:1009–13. doi: 10.1001/archinte.160.7.1009 10761967

[4] Szczuka D Nowak A Zakłos-Szyda M Kochan E Szymańska G Motyl I . American ginseng (*Panax quinquefolium* L.) as a source of bioactive phytochemicals with pro-health properties. Nutrients. (2019) 11:1041. doi: 10.3390/nu11051041, 31075951 PMC6567205

[5] Nag SA Qin JJ Wang W Wang MH Wang H Zhang R. Ginsenosides as anticancer agents: in vitro and in vivo activities, structure–activity relationships, and molecular mechanisms of action. Front Pharmacol. (2012) 3:25. doi: 10.3389/fphar.2012.00025, 22403544 PMC3289390

[6] Hui J Wang Y Xu F Zhao J. Potential preventive effects of selected traditional Chinese medicine as adjuvant therapy on hypertensive heart disease progression by replenishing qi and activating blood circulation: a systematic review and meta-analysis of clinical trials. Front Pharmacol. (2025) 16:1506234. doi: 10.3389/fphar.2025.1506234, 41111519 PMC12528026

[7] Liu Y Wang Z Zhang X Chen H Ruan D Bai S . Characterization of volatile substances in Pu-erh tea (raw tea) at different storage times. Foods. (2025) 14:840. doi: 10.3390/foods14050840, 40077543 PMC11898881

[8] Lv HP Zhang YJ Lin Z Liang YR. Processing and chemical constituents of Pu-erh tea: a review. Food Res Int. (2013) 53:608–18. doi: 10.1016/j.foodres.2013.02.043

[9] Wang S Qiu Y Gan RY Zhu F. Chemical constituents and biological properties of Pu-erh tea. Food Res Int. (2022) 154:110899. doi: 10.1016/j.foodres.2021.110899, 35337597

[10] Deng YT Lin-Shiau SY Shyur LF Lin JK. Pu-erh tea polysaccharides decrease blood sugar by inhibition of α-glucosidase activity in vitro and in mice. Food Funct. (2015) 6:1539–46. doi: 10.1039/C4FO01025F, 25820466

[11] Zeng L Yan J Luo L Zhang D. Effects of Pu-erh tea aqueous extract (PTAE) on blood lipid metabolism enzymes. Food Funct. (2015) 6:2008–16. doi: 10.1039/C5FO00362H 26018873

[12] Gupta A Sanwal N Bareen MA Barua S Sharma N Olatunji OJ . Trends in functional beverages: functional ingredients, processing technologies, stability, health benefits, and consumer perspective. Food Res Int. (2023) 170:113046. doi: 10.1016/j.foodres.2023.113046 37316029

[13] Ni Z Zhao X Yu P Huang H Bao X Li J . Characterization of the sensory properties and quality components of Huangjin green tea based on molecular sensory-omics. Foods. (2023) 12:3234. doi: 10.3390/foods12173234 PMC10486783 37685167

[14] Yue C Wang Z Peng H Jiang L Yang P Li W. Analysis of taste quality differences between high- and low-grade Ninghong tea: from the perspective of sensory evaluation, metabolomics, and taste activity values. Foods. (2024) 13:3957. doi: 10.3390/foods13233957 PMC11641024 39683030

[15] Lu L Liu J Zhang W Cheng X Zhang B Yang Y . Key factors of quality formation in Wuyi black tea during processing timing. Foods. (2024) 13:1373. doi: 10.3390/foods13091373, 38731743 PMC11083133

[16] Zhou M Deng X Wang Q Wei Z Wang X Yuan W . Flavor characteristics of sun-dried green tea in different regions of Yunnan: metabolite basis and soil influencing factors. Foods. (2025) 14:1280. doi: 10.3390/foods14071280, 40238592 PMC11988626

[17] Nian B Chen L Yi C Shi X Jiang B Jiao W . A high performance liquid chromatography method for simultaneous detection of 20 bioactive components in tea extracts. Electrophoresis. (2019) 40:2837–44. doi: 10.1002/elps.201900154, 31353482

[18] Jin G Bi C Ji A Hu J Zhang Y Yang L . Volatile profiling of Tongcheng Xiaohua tea from different geographical origins: a multimethod investigation using sensory analysis, electronic nose, HS-SPME-GC-MS, and chemometrics. Foods. (2025) 14:1996. doi: 10.3390/foods14111996 PMC12155478 40509523

[19] Li H Geng W Haruna SA Zhou C Wang Y Ouyang Q . Identification of characteristic volatiles and metabolomic pathway during pork storage using HS-SPME-GC/MS coupled with multivariate analysis. Food Chem. (2022) 373:131431. doi: 10.1016/j.foodchem.2021.131431, 34700034

[20] Sun C Wang R Wang T Li Q. Primary evaluation of nine volatile N-nitrosamines in raw red meat from Tianjin, China by HS-SPME-GC-MS. Food Chem. (2020) 310:125945. doi: 10.1016/j.foodchem.2019.125945 31837529

[21] Hou Z Chen Z Li L Chen H Zhang H Liu S . Comparison of volatile compounds in Jingshan green tea scented with different flowers using GC-IMS and GC-MS analyses. Foods. (2024) 13:2653. doi: 10.3390/foods13172653, 39272418 PMC11394657

[22] Want EJ O'Maille G Smith CA Brandon TR Uritboonthai JC Qin C . Development of a robust and repeatable UPLC-MS method for the long-term metabolomic study of human serum. Anal Chem. (2009) 81:1357–64. doi: 10.1021/ac8019366 19170513

[23] Want EJ Masson P Michopoulos F Wilson ID Theodoridis G Plumb RS . Global metabolic profiling of animal and human tissues via UPLC-MS. Nat Protoc. (2013) 8:17–32. doi: 10.1038/nprot.2012.135, 23222455

[24] Chen C Wu Y Li J Wang X Zeng Z Xu J . TBtools-II: a “one for all, all for one” bioinformatics platform for biological big-data mining. Mol Plant. (2023) 16:1733–42. doi: 10.1016/j.molp.2023.09.010, 37740491

[25] Zhang L Cao QQ Granato D Xu YQ Ho CT. Association between chemistry and taste of tea: a review. Trends Food Sci Technol. (2020) 101:139–49. doi: 10.1016/j.tifs.2020.05.015

[26] Wu R Liang H Hu N Lu J Li C Tang D. Chemical, sensory variations in black teas from six tea cultivars in Jingshan, China. Foods. (2025) 14:1558. doi: 10.3390/foods14091558, 40361640 PMC12071611

[27] Huang W Liu Q Ning J. Effect of tea stems on the quality formation of large-leaf yellow tea: sensomics and flavoromics approaches. Food Chem X. (2024) 24:101794. doi: 10.1016/j.fochx.2024.101794, 39290754 PMC11406333

[28] Zeng Q Wang H Tuo J Ding Y Cao H Yue C. Volatile organic compounds in teas: identification, extraction, analysis, and application of tea aroma. Foods. (2025) 14:2574. doi: 10.3390/foods14152574, 40807511 PMC12346385

[29] Deng X He G Tan Q Li X Wang B Wang X. Evolution analysis of flavor-active compounds during artificial fermentation of Pu-erh tea. Food Chem. (2021) 357:129783. doi: 10.1016/j.foodchem.2021.129783 33892356

[30] Wang X Yu W Cao C Yuan X Qiu J Kong F . Comparison of potent odorants in raw and ripened Pu-erh tea infusions based on odor activity value calculation and multivariate analysis: understanding the role of pile fermentation. J Agric Food Chem. (2019) 67:13139–49. doi: 10.1021/acs.jafc.9b05321 31631665

[31] Li Y Wu T Deng X Tian D Ma C Wang X . Characteristic aroma compounds in naturally withered and combined-withered γ-aminobutyric acid white tea revealed by HS-SPME-GC-MS and relative odor activity value. LWT. (2023) 176:114467. doi: 10.1016/j.lwt.2023.114467

[32] Sumner LW Amberg A Barrett D Beale MH Beger R Daykin CA . Proposed minimum reporting standards for chemical analysis: chemical analysis working group (CAWG) metabolomics standards initiative (MSI). Metabolomics. (2007) 3:211–21. doi: 10.1007/s11306-007-0082-2, 24039616 PMC3772505

[33] Ge Y Bian X Sun B Zhao M Ma Y Tang Y . Dynamic profiling of phenolic acids during Pu-erh tea fermentation using derivatization liquid chromatography–mass spectrometry approach. J Agric Food Chem. (2019) 67:4568–77. doi: 10.1021/acs.jafc.9b00789, 30932482

[34] Scharbert S Holzmann N Hofmann T. Identification of the astringent taste compounds in black tea infusions by combining instrumental analysis and human bioresponse. J Agric Food Chem. (2004) 52:3498–508. doi: 10.1021/jf049802u 15161222

[35] Scharbert S Hofmann T. Molecular definition of black tea taste by means of quantitative studies, taste reconstitution, and omission experiments. J Agric Food Chem. (2005) 53:5377–84. doi: 10.1021/jf050294d, 15969522

[36] Chen Y Liao Z Wang Z Shi W Xu J. Isolation and identification of bitter compounds in ginseng (*Panax ginseng* C. A. Mey.) based on preparative high-performance liquid chromatography, UPLC-Q-TOF/MS, and electronic tongue. Separations (Basel). (2024) 11:114. doi: 10.3390/separations11040114

[37] Chen YH Zhang YH Chen GS Yin JF Chen JX Wang F . Effects of phenolic acids and quercetin-3-O-rutinoside on the bitterness and astringency of green tea infusion. NPJ Sci Food. (2022) 6:8. doi: 10.1038/s41538-022-00124-8, 35087059 PMC8795203

[38] Wu T Wang X Wang X He J Yuan W Che R . Metabolomic and flavoromic insights into the effects of Polygonatum kingianum extract during black tea processing. Front Nutr. (2026) 13:1882990. doi: 10.3389/fnut.2026.1882990, 42488214 PMC13388378

[39] Guo J Yu Z Liu M Guan M Shi A Hu Y . Analysis of volatile profile and aromatic characteristics of raw Pu-erh tea during storage based on GC-MS and odor activity value. Foods. (2023) 12:3568. doi: 10.3390/foods12193568, 37835224 PMC10572200

[40] Ma L Gao M Hu J Tong W Du L Yu A . Characterization of the key active aroma compounds in Pu-erh tea using gas chromatography–time-of-flight/mass spectrometry–olfactometry combined with five different evaluation methods. Eur Food Res Technol. (2022) 248:45–56. doi: 10.1007/s00217-021-03847-2

[41] Szymańska E Saccenti E Smilde AK Westerhuis JA. Double-check: validation of diagnostic statistics for PLS-DA models in metabolomics studies. Metabolomics. (2012) 8:3–16. doi: 10.1007/s11306-011-0330-3, 22593721 PMC3337399

[42] Yang Z Xie Y Zhu Y Lei M Chen X Jin W . Unraveling the flavor formation process of mellow- and thick-type ripened Pu-erh tea through non-targeted metabolomics and metagenomics. Food Chem X. (2025) 27:102424. doi: 10.1016/j.fochx.2025.102424, 40241696 PMC12002954

[43] Xie G Ye M Wang Y Ni Y Su M Huang H . Characterization of Pu-erh tea using chemical and metabolic profiling approaches. J Agric Food Chem. (2009) 57:3046–54. doi: 10.1021/jf804000y, 19320437

[44] Xu Y Liang PL Chen XL Gong MJ Zhang L Qiu XH . The impact of citrus–tea cofermentation process on chemical composition and contents of Pu-erh tea: an integrated metabolomics study. Front Nutr. (2021) 8:737539. doi: 10.3389/fnut.2021.737539, 34604284 PMC8484324

[45] Geng X Wang J Liu Y Liu L Liu X Zhao Y . Research progress on chemical diversity of saponins in *Panax ginseng*. Chin Herb Med. (2024) 16:529–47. doi: 10.1016/j.chmed.2024.08.005, 39606259 PMC11589341

[46] Fan J Liu F Ji W Wang X Li L. Comprehensive investigation of ginsenosides in steamed *Panax quinquefolius* under different processing conditions using LC-MS. Molecules. (2024) 29:623. doi: 10.3390/molecules29030623 PMC10856252 38338369

[47] van Gemert LJ. Odour Thresholds: Compilations of Odour Threshold Values in Air, Water, and Other Media. 2nd enlarged and revised ed. Utrecht: Oliemans Punter & Partners BV (2011).

[48] Mu S Stieger M Boesveldt S. Olfactory discrimination of fat content in milks is facilitated by differences in volatile compound composition rather than odor intensity. Food Chem. (2022) 393:133357. doi: 10.1016/j.foodchem.2022.133357, 35667180

[49] Baker RA. Threshold odors of organic chemicals. J Am Water Works Assoc. (1963) 55:913–6. doi: 10.1002/j.1551-8833.1963.tb01102.x

[50] Czerny M Christlbauer M Christlbauer M Fischer A Granvogl M Hammer M . Re-investigation on odour thresholds of key food aroma compounds and development of an aroma language based on odour qualities of defined aqueous odorant solutions. Eur Food Res Technol. (2008) 228:265–73. doi: 10.1007/s00217-008-0931-x

[51] Neta ERD Miracle RE Sanders TH Drake MA. Characterization of alkylmethoxypyrazines contributing to earthy/bell pepper flavor in farmstead cheddar cheese. J Food Sci. (2008) 73:C632–8. doi: 10.1111/j.1750-3841.2008.00948.x 19021793

[52] Rashash DMC Dietrich AM Hoehn RC. FPA of selected odorous compounds. J Am Water Works Assoc. (1997) 89:131–41. doi: 10.1002/j.1551-8833.1997.tb08213.x

[53] Giri A Osako K Okamoto A Ohshima T. Olfactometric characterization of aroma-active compounds in fermented fish paste in comparison with fish sauce, fermented soy paste and sauce products. Food Res Int. (2010) 43:1027–40. doi: 10.1016/j.foodres.2010.01.012

[54] Buttery RG Teranishi R Ling LC Turnbaugh JG. Quantitative and sensory studies on tomato paste volatiles. J Agric Food Chem. (1990) 38:336–40. doi: 10.1021/jf00091a074

[55] Wang K Xiao Y Xie N Xu H Li S Liu C . Effect of leaf grade on taste and aroma of shaken Hunan black tea. Foods. (2023) 13:42. doi: 10.3390/foods13010042, 38201072 PMC10778213

[56] Khiari D Brenner L Burlingame GA Suffet IH. Sensory gas chromatography for evaluation of taste and odor events in drinking water. Water Sci Technol. (1992) 25:97–104. doi: 10.2166/wst.1992.0040

[57] Buttery RG Turnbaugh JG Ling LC. Contribution of volatiles to rice aroma. J Agric Food Chem. (1988) 36:1006–9. doi: 10.1021/jf00083a025

