# Supplementary material for: Integrated flavoromics and metabolomics characterize flavor and chemical composition differences in American ginseng–ripe Pu-erh tea blends
Source: Front Nutr. 2026 Sep 28;13:1963132. doi: 10.3389/fnut.2026.1963132 (PMC13655095; doi:10.3389/fnut.2026.1963132)
Supplement: Supplementary file 1 [file Supplementary_file_1.docx]

Supplementary files

**Supplementary Table S1. Response characteristics of the PEN3 electronic-nose sensor array.**

| Sensor | Response characteristics |
| --- | --- |
| W1C | Sensitive to aromatic compounds, particularly benzene derivatives |
| W5S | Highly sensitive to nitrogen oxides |
| W3C | Sensitive to aromatic compounds and ammonia |
| W6S | Selective for hydrogen |
| W5C | Sensitive to short-chain alkanes and aromatic compounds |
| W1S | Sensitive to methyl-containing compounds |
| W1W | Sensitive to sulfur-containing compounds |
| W2S | Sensitive to alcohols, aldehydes, and ketones |
| W2W | Sensitive to aromatic compounds and organic sulfur compounds |
| W3S | Sensitive to long-chain alkanes |

**Note: The PEN3 electronic-nose system is equipped with 10 metal oxide semiconductor (MOS) sensors. The response characteristics were summarized according to the manufacturer’s specifications and published literature. These descriptions indicate the principal response tendencies of the sensors rather than absolute selectivity. Because MOS sensors exhibit cross-sensitivity to multiple volatile compounds, the response of an individual sensor cannot be directly attributed to a specific compound or odor attribute.**

**Supplementary Table S2. Sensory evaluation results for different American ginseng materials, ripe Pu-erh tea, and their blends.**

| Sample | Appearance（10%） | | Liquor color（20%） | | Aroma（35%） | | Taste（35%） | | Overall sensory score |
| --- | --- | --- | --- | --- | --- | --- | --- | --- | --- |
|  | Sensory comments | Score | Sensory comments | Score | Sensory comments | Score | Sensory comments | Score |  |
| RP | Dark brown, uniform, fine-textured, and clean | 85.0 ± 1.00ᵈᵉ | Deep red, bright | 93.0 ± 0.71ᵃ | Aged aroma | 92.0 ± 1.22ᵃᵇ | Full-bodied, mellow, and smooth | 88.0 ± 1.00ᵃᵇ | 90.1± 0.27ᵇ |
| WS | Pale yellowish-white, uniform, fine-textured, and clean | 86.0 ± 0.71ᶜᵈ | Deep yellow | 88.0 ± 0.71ᶜ | Ginseng aroma and chrysanthemum-like aroma | 88.0 ± 1.00ᶜᵈ | Mellow and full-bodied, slightly astringent, with ginseng and chrysanthemum-like notes | 86.0 ± 0.71ᵇᶜ | 87.1± 0.13ᵈ |
| DSX | Yellowish-brown, relatively uniform, relatively fine-textured, and clean | 85.0 ± 0.71ᵈᵉ | Apricot yellow | 87.0 ± 1.00ᶜ | Pronounced ginseng aroma with a fresh citrus note | 86.0 ± 0.71ᵈᵉ | Rich and full-bodied, slightly astringent, with a pronounced ginseng taste, sweet aftertaste, and salivating sensation | 86.0 ± 0.71ᵇᶜ | 86.1 ± 0.13ᵉ |
| DSD | Yellowish-brown, relatively uniform, relatively fine-textured, and clean | 84.0 ± 0.71ᵉ | Light yellow | 85.0 ± 1.00ᵈ | Intense ginseng aroma with a slight medicinal note | 84.0 ± 1.00ᵉ | Relatively light, moderately astringent, with a noticeable ginseng taste, sweet aftertaste, and salivating sensation | 83.0 ± 1.22ᵈᵉ | 83.9± 0.30ʰ |
| SP1 | Slightly variegated with pale yellowish-white tones, uniform, and clean | 87.0 ± 0.71ᵇᶜ | Brownish-red, lacking brightness | 83.0 ± 0.71ᵉ | Intense aged aroma with a pronounced ginseng note | 88.0 ± 1.00ᶜᵈ | Fairly mellow, with a pronounced ginseng taste, particulate sensation, and rough mouthfeel | 84.0 ± 1.22ᶜᵈ | 85.5 ± 0.28ᶠ |
| SP2 | Relatively bright in color, fine, uniform, and clean | 89.0 ± 0.71ᵃ | Light red, relatively bright | 84.0 ± 0.71ᵈᵉ | Aged aroma with a chrysanthemum-like note | 90.0 ± 1.00ᵇᶜ | Mellow and full-bodied, with a ginseng taste and a pronounced sweet aftertaste | 89.0 ± 1.00ᵃ | 88.4 ± 0.23ᶜ |
| SP3 | Slightly variegated with brown tones, uniform, and clean | 88.0 ± 0.71ᵃᵇ | Red, fairly bright | 85.0 ± 1.00ᵈ | Aged aroma and ginseng aroma | 86.0 ± 1.00ᵈᵉ | Smooth, with a chrysanthemum-like note and a slight stuffy cooked note | 87.0 ± 1.22ᵃᵇ | 86.4 ± 0.37ᵉ |
| SP4 | Dark-colored, uniform, fine-textured, and clean | 89.0 ± 0.71ᵃ | Orange-red, bright | 91.0 ± 1.00ᵇ | Sweet orange-like aroma | 93.0 ± 1.22ᵃ | Mellow and full-bodied, with a rich and pronounced taste | 89.0 ± 0.71ᵃ | 90.8 ± 0.23ᵃ |
| SP5 | Dark brown, relatively uniform, relatively fine-textured, and clean | 88.0 ± 1.00ᵃᵇ | Deep red, relatively bright | 88.0 ± 0.71ᶜ | Aged aroma with a ginseng note | 86.0 ± 1.00ᵈᵉ | Mellow and full-bodied, with a mild ginseng taste and sweet aftertaste | 83.0 ± 0.71ᵈᵉ | 85.6 ± 0.19ᶠ |
| SP6 | Dark brown, uniform, relatively fine-textured, and relatively clean | 87.0 ± 1.00ᵇᶜ | Red, bright | 87.0 ± 1.00ᶜ | Aged aroma with a slight ginseng note | 85.0 ± 1.00ᵉ | Relatively thick and rich in taste, with noticeable astringency | 83.0 ± 0.71ᵈᵉ | 84.9 ± 0.23ᵍ |
| SP7 | Slightly variegated with brown tones, relatively uniform, and clean | 86.0 ± 0.71ᶜᵈ | Red, relatively bright | 85.0 ± 1.00ᵈ | Aged aroma with pronounced complex aromatic notes | 84.0 ± 1.00ᵉ | Somewhat dull in taste, strongly astringent, and slightly bitter | 81.0 ± 1.00ᵉ | 83.4± 0.23ʰ |

**Note: Sensory evaluation was conducted according to GB/T 23776-2018 by five assessors (n = 5). Each attribute was scored on a 100-point scale, and scores are presented as mean ± standard deviation (SD). The overall sensory score was calculated as appearance × 10% + liquor color × 20% + aroma × 35% + taste × 35%. Within each screening stage, scores for each attribute were analyzed by one-way analysis of variance followed by Tukey’s honestly significant difference test. Different lowercase superscript letters within the same scoring column indicate significant differences among samples included in that comparison (P < 0.05). Sensory comments summarize the descriptors agreed upon by the panel. Sample definitions and compositions are provided in Section 2.1. SP4 was included as a commercial reference sample.**

**Supplementary Table S3. Physicochemical properties of different American ginseng materials, ripe Pu-erh tea, and their blends.**

| Sample | Moisture Content (%) | Water Extract (%) | Total Tea Polyphenols (%) | Soluble Sugars (%) | Total Flavonoids (%) | Free Amino Acids (%) |
| --- | --- | --- | --- | --- | --- | --- |
| RP | 8.87 ± 0.012 | 43.17 ± 0.066 | 14.56 ± 0.472 | 7.00 ± 0.689 | 13.85 ± 0.118 | 0.95 ± 0.046 |
| WS | 7.58 ± 0.023 | 52.83 ± 0.281 | 4.68 ± 0.228 | 9.16 ± 0.489 | 19.79 ± 0.878 | 1.61 ± 0.101 |
| DSX | 9.74 ± 0.055 | 59.22 ± 0.126 | 2.34 ± 0.744 | 10.15 ± 0.436 | 1.48 ± 0.321 | 1.00 ± 0.005 |
| DSD | 6.92 ± 0.064 | 64.68 ± 0.450 | 1.34 ± 0.335 | 13.69 ± 0.853 | 1.22 ± 0.356 | 2.10 ± 0.051 |
| SP1 | 8.29 ± 0.084 | 46.58 ± 0.809 | 12.16 ± 0.013 | 13.19 ± 0.337 | 16.60 ± 0.058 | 1.24 ± 0.178 |
| SP2 | 8.65 ± 0.062 | 45.68 ± 0.123 | 10.63 ± 0.700 | 9.90 ± 0.656 | 14.50 ± 0.534 | 1.03 ± 0.263 |
| SP3 | 9.21 ± 0.031 | 43.55 ± 0.209 | 12.88 ± 0.001 | 9.12 ± 0.101 | 14.41 ± 0.673 | 0.97 ± 0.025 |
| SP4 | 5.97 ± 0.082 | 49.07 ± 0.140 | 10.63 ± 0.559 | 14.80 ± 0.666 | 14.40 ± 0.893 | 1.09 ± 0.049 |
| SP5 | 8.77 ± 0.075 | 44.59 ± 0.224 | 12.95 ± 0.632 | 7.45 ± 0.653 | 15.06 ± 0.551 | 1.10 ± 0.014 |
| SP6 | 9.09 ± 0.082 | 45.60 ± 0.506 | 14.52 ± 0.411 | 9.75 ± 0.468 | 13.01 ± 0.334 | 0.95 ± 0.030 |
| SP7 | 8.50 ± 0.055 | 45.62 ± 0.455 | 13.99 ± 0.720 | 11.03 ± 0.512 | 11.74 ± 0.810 | 1.03 ± 0.027 |

**Note: Values are presented as mean ± standard deviation (SD) of three replicate determinations for each sample (n = 3). Except for moisture content, all physicochemical values are expressed on a dry-weight basis. Sample codes and blend compositions are defined in Section 2.1 and Table 2. SP4 is a commercial reference product.**

**Supplementary Table S4. Tentatively identified candidate aroma-related compounds in American ginseng, ripe Pu-erh tea, and their blended samples.**

| No. | Tentatively identified compound | CAS | RIexp | RIlit | ΔRI | Class | Odor description | Odor threshold used (mg kg−1) | Threshold matrix | Threshold source | RP | WS | SP1 | SP2 | SP3 | SP4 | P value | VIP (Comp. 1) |
| --- | --- | --- | --- | --- | --- | --- | --- | --- | --- | --- | --- | --- | --- | --- | --- | --- | --- | --- |
| 1 | Heptanal | 111-71-7 | 1196.60 | 1185.00 | 11.60 | Aldehydes | Citrus, fatty, rancid | 0.003 | Water | van Gemert (2011) ^[47]^ | 4.83 | 8.67 | 7.81 | 7.50 | 5.33 | 8.68 | 0.04 | 0.81 |
| 2 | 2-Undecanone | 112-12-9 | 1612.25 | 1598.00 | 14.25 | Ketones | Orange, fresh, green | 0.0055 | Water | van Gemert (2011) ^[47]^；Mu et al. (2022) ^[48]^ | 5.91 | 2.76 | 7.67 | 2.93 | 9.32 | 4.77 | 0.01 | 0.55 |
| 3 | Ethyl acrylate | 140-88-5 | 1004.20 | 992.00 | 12.20 | Esters | Sweet, ester-like, plastic, sharp | 0.0067 | Water | van Gemert (2011) ^[47]^;Baker (1963) ^[49]^ | 0.28 | 0.00 | 0.12 | 0.12 | 0.12 | 0.00 | 0.00 | 1.78 |
| 4 | Trimethylpyrazine | 14667-55-1 | 1411.93 | 1402.00 | 9.93 | Organoheterocyclic compounds | Roasted nuts, cocoa, peanuts | 0.023 | Water | van Gemert (2011) ^[47]^ | 0.86 | 0.95 | 0.86 | 0.87 | 0.70 | 0.31 | 0.00 | 1.28 |
| 5 | (E)-2-Nonenal | 18829-56-6 | 1552.00 | 1534.00 | 18.00 | Aldehydes | Fatty, cucumber | 0.00019 | Water | Czerny et al. (2008) ^[50]^ | 100.00 | 100.00 | 100.00 | 100.00 | 100.00 | 100.00 | 0.80 | 0.82 |
| 6 | 2-Methoxy-3-(1-methylpropyl)pyrazine | 24168-70-5 | 1520.00 | 1502.00 | 18.00 | Organoheterocyclic compounds | — | 0.000001 | Water | Neta et al. (2008) ^[51]^ | 0.33 | 1.47 | 0.99 | 0.80 | 0.55 | 0.66 | 0.00 | 1.40 |
| 7 | (E)-2-Octenal | 2548-87-0 | 1446.20 | 1429.00 | 17.20 | Aldehydes | Nutty, green, fatty | 0.003 | Water | van Gemert (2011) ^[47]^ | 6.46 | 5.67 | 6.20 | 5.90 | 6.20 | 5.62 | 0.00 | 1.48 |
| 8 | 2-Pentylfuran | 3777-69-3 | 1242.90 | 1232.00 | 10.90 | Organoheterocyclic compounds | Green beans, vegetable | 0.006 | Water | van Gemert (2011) ^[47]^ | 4.64 | 4.73 | 3.57 | 3.86 | 2.73 | 3.36 | 0.69 | 0.56 |
| 9 | 2,3-Butanedione | 431-03-8 | 989.50 | 979.00 | 10.50 | Ketones | Pleasant, buttery | 0.001 | Water | Czerny et al. (2008) ^[50]^ | 3.51 | 7.90 | 4.79 | 4.23 | 6.16 | 8.09 | 0.18 | 0.92 |
| 10 | 1-Octen-3-one | 4312-99-6 | 1314.30 | 1301.00 | 13.30 | Ketones | Mushroom-like | 0.000016 | Water | Czerny et al. (2008) ^[50]^ | 2.71 | 3.59 | 3.61 | 3.33 | 3.67 | 3.54 | 0.00 | 0.26 |
| 11 | 3-Methylbutanoic acid | 503-74-2 | 1684.00 | 1666.00 | 18.00 | Fatty acyls | Rancid cheese, sweaty, putrid | 0.02 | Water | van Gemert (2011) ^[47]^; Rashash et al. (1997) ^[52]^ | 0.22 | 0.53 | 0.50 | 0.45 | 0.33 | 0.39 | 0.25 | 0.60 |
| 12 | (E,Z)-2,6-Nonadienal | 557-48-2 | 1603.20 | 1584.00 | 19.20 | Aldehydes | Cucumber, green | 0.0000045 | Water | Czerny et al. (2008) ^[50]^ | 0.68 | 0.51 | 0.65 | 0.92 | 0.27 | 0.62 | 0.50 | 0.73 |
| 13 | 2-Methylbutanal | 96-17-3 | 921.10 | 914.00 | 7.10 | Aldehydes | Cocoa, almond | 0.001 | Water | van Gemert (2011) ^[47]^; Giri et al. (2010) ^[53]^ | 8.54 | 21.97 | 10.22 | 11.03 | 7.38 | 12.72 | 0.00 | 1.52 |
| 14 | Pentanal | 110-62-3 | 989.50 | 979.00 | 10.50 | Aldehydes | Sickening, rancid, decayed | 0.012 | Water | Buttery et al. (1990) ^[54]^ | 0.00 | 0.12 | 0.00 | 0.00 | 0.00 | 0.00 | 0.00 | 1.11 |
| 15 | (E)-2-Dodecenal | 20407-84-5 | 1877.30 | 1867.00 | 10.30 | Aldehydes | Green, fatty, sweet | 0.0014 | Water | Wang et al. (2023) ^[55]^ | 0.00 | 1.57 | 1.87 | 0.93 | 0.43 | 1.99 | 0.13 | 0.75 |
| 16 | Ethyl palmitate | 628-97-7 | 2257.22 | 2251.00 | 6.22 | Fatty acyls | Waxy | 2 | Water | van Gemert (2011) ^[47]^ | 0.00 | 0.10 | 0.17 | 0.12 | 0.11 | 0.00 | 0.00 | 0.12 |
| 17 | Acetic acid | 64-19-7 | 1464.53 | 1449.00 | 15.53 | Carboxylic acids | Pungent, vinegar-like | 99 | Water | Czerny et al. (2008) ^[50]^ | 0.00 | 0.15 | 0.14 | 0.10 | 0.00 | 0.00 | 0.00 | 1.06 |
| 18 | α-Pinene | 80-56-8 | 1029.20 | 1027.00 | 2.20 | Lipids and lipid-like molecules | Turpentine-like, resinous, pine-like | 0.014 | Water(racemic α-pinene) | Czerny et al. (2008) ^[50]^ | 0.00 | 0.47 | 0.23 | 0.14 | 0.00 | 0.00 | 0.00 | 0.94 |
| 19 | 2-Ethyl-1-hexanol | 104-76-7 | 1496.44 | 1491.00 | 5.44 | Alcohols | Rose, green | 0.198 | Water | van Gemert (2011) ^[47]^ Khiari et al. (1992) ^[56]^ | 0.00 | 0.00 | 0.12 | 0.13 | 0.12 | 0.00 | 0.15 | 1.04 |
| 20 | Phenylacetaldehyde | 122-78-1 | 1660.00 | 1641.00 | 19.00 | Aldehydes | Floral, honey-like | 0.004 | Water | Buttery et al. (1988) ^[57]^ | 0.00 | 0.00 | 0.10 | 0.00 | 0.00 | 0.00 | 0.34 | 0.27 |

**Note: CAS denotes the Chemical Abstracts Service registry number. RIexp and RIlit denote the experimental and literature retention indices, respectively, and ΔRI is their absolute difference. Because authentic standards were not used for confirmation, all listed compounds were regarded as tentatively identified. Odor descriptions, odor thresholds, threshold matrices, and threshold sources were obtained from the cited literature. ROAV denotes relative odor activity value; the values in the RP, WS, and SP1–SP4 columns are the ROAVs of the corresponding compounds. As the odor thresholds used were determined in water and may differ from those in tea infusions, these ROAVs were used only to estimate potential relative aroma contributions. Compounds with ROAV ≥ 1 were regarded as potential key aroma-active candidates, and those with 0.10 ≤ ROAV < 1 as potential aroma-modifying candidates. P values are nominal, unadjusted values from one-way analysis of variance across sample groups; P < 0.05 indicates a nominal statistical difference. Because no correction for multiple testing was applied, these results should be interpreted as exploratory. VIP (Comp. 1) denotes the variable importance in projection value for the first component of the OPLS-DA model. Sample definitions are provided in Figure 1.**

**Supplementary Figure S1. Hierarchical clustering heatmaps of nine classes of differential non-volatile metabolite features.**

**The differential metabolite features shown in the figure comprise nine categories: flavonoids; terpenoids and prenol lipids; phenols and phenylpropanoids; alkaloids and N-containing compounds; organooxygen compounds and saccharides; benzene and aromatic derivatives; organic acids and derivatives; lipids and fatty acyls; and others. The heatmaps were generated using the mean relative signal intensities obtained from three replicate measurements (n = 3), followed by row-wise Z-score standardization. Purple and blue indicate relatively higher and lower signal intensities, respectively, whereas lighter colors indicate values closer to the mean. The dendrograms show the hierarchical clustering of metabolite features. RP, ripe Pu-erh tea powder; WS, Wendeng American ginseng powder; SP1, 65% RP + 35% WS; SP2, 75% RP + 25% WS; SP3, 85% RP + 15% WS; SP4, a commercial granulated-tea reference sample containing American ginseng (≥15%), ripe Pu-erh tea, chicory root, and chrysanthemum. Because the analysis was based on relative mass spectrometric signal intensities and the annotations were not confirmed using authentic standards, the heatmaps represent the relative abundance patterns of tentatively annotated metabolite features rather than their absolute concentrations.**
